# Pressure flaking to serrate bifacial points for the hunt during the MIS5 at Sibudu Cave (South Africa)

**DOI:** 10.1371/journal.pone.0175151

**Published:** 2017-04-26

**Authors:** Veerle Rots, Carol Lentfer, Viola C. Schmid, Guillaume Porraz, Nicholas J. Conard

**Affiliations:** 1 Chercheur Qualifié du FNRS, TraceoLab / Prehistory, University of Liège, Liège, Belgium; 2 TraceoLab / Prehistory, University of Liège, Liège, Belgium; 3 Department of Early Prehistory and Quaternary Ecology, Eberhard Karls Universität Tübingen, Tübingen, Germany; 4 UMR 7041, Equipe AnTET, Université Paris Ouest Nanterre La Défense, Nanterre Cedex, France; 5 CNRS, UMR 7041, Equipe AnTET, Université Paris Ouest Nanterre La Défense, Nanterre, France; 6 Evolutionary Studies Institute, University of the Witwatersrand, Johannesburg, South Africa; 7 Senckenberg, Center for Human Evolution and Paleoenvironment, Eberhard Karls Universität Tübingen, Tübingen, Germany; Max Planck Institute for the Science of Human History, GERMANY

## Abstract

Projectile technology is considered to appear early in the southern African Middle Stone Age (MSA) and the rich and high resolution MSA sequence of Sibudu Cave in KwaZulu-Natal has provided many new insights about the use and hafting of various projectile forms. We present the results of a functional and technological analysis on a series of unpublished serrated bifacial points recently recovered from the basal deposits of Sibudu Cave. These serrated tools, which only find equivalents in the neighbouring site of Umhlatuzana, precede the Still Bay techno-complex and are older than 77 ka BP. Independent residue and use-wear analyses were performed in a phased procedure involving two separate analysts, which allowed the engagement between two separate lines of functional evidence. Thanks to the excellent preservation at Sibudu Cave, a wide range of animal, plant and mineral residues were observed in direct relation with diagnostic wear patterns. The combination of technological, wear and residue evidence allowed us to confirm that the serration was manufactured with bone compressors and that the serrated points were mounted with a composite adhesive as the tips of projectiles used in hunting activities. The suite of technological and functional data pushes back the evidence for the use of pressure flaking during the MSA and highlights the diversity of the technical innovations adopted by southern African MSA populations. We suggest the serrated points from the stratigraphic units Adam to Darya of Sibudu illustrate one important technological adaptation of the southern African MSA and provide another example of the variability of MSA bifacial technologies.

## Introduction

The South African Middle Stone Age (MSA) surprises by the multiplicity of the archaeological discoveries, which may be attributed in part to the research intensity in this part of the world and partly to the unique nature of the landscape and human behaviors. Today, the South African MSA has become an ideal canvas for the development and elaboration of models helping us to understand the first societies of anatomically modern humans (AMH), before their dispersal on the Eurasian continent. Important sites such as those of Klasies River main site (KRM), Blombos Cave (BBC), Diepkloof Rock Shelter (DRS), Pinnacle Point (PP) and also Sibudu Cave have been instrumental to scientific research and model building. The purpose of this article is to present new evidence as testament to the originality of the behavioral traits that characterize the South African MSA. Our study focuses on the newly excavated MIS5 levels of the site of Sibudu Cave.

Technical innovations are important testimonials of the evolution of human societies since they have the potential to reflect new adaptations of societies to their environment, new social and economic organizations, as well as differences in cognitive architectures [1,2]. Technology itself, involves more than the mere production of stone tools, but the stone tools are nevertheless an essential basis on which to rely for understanding of the past.

The early appearance of thermal treatment for improving stone knapping qualities [3,4], the use of pressure for the shaping of bifacial points [5–7] and also, the appearance of geometric tools requiring different ways of manipulation during use and new hafting modes [8,9] are some examples of technical innovations that characterize the South-African MSA. These innovations are mostly concentrated in two particular technocomplexes–the Still Bay (SB) and the Howiesons Poort (HP)–where there is also rich evidence for symbolic and visual communication in the form of engravings on ochre and ostrich eggshell [10,11] and other ornamental expressions [12]. Nevertheless, the SB and the HP technocomplexes cannot be assumed to represent the specifics of the South African MSA in its entirety. It is therefore essential that we examine older phases leading up to the SB to better contextualize and understand the nature, development and significance of these technical innovations.

Questions about the timing and the type of projectile points in the history of hunter-gatherer societies comes from fundamental research on the history of techniques and are the subject of a rich literature and debate. Elements under discussion touch upon aspects of the recognition of functional stigmata (e.g., experimentation, analysts and analyses) but also of the implications of certain interpretations (e.g., type of hunting equipment/projecting mode). In the South African Stone Age, the presence of projectile points has been proposed for the site of Kathu Pan dated to 500 ka BP [13], but serious doubts have been raised regarding the reliability of this identification [14] and the association between the tools and the dated geological horizon [2]. During the Howiesons Poort, an important change may have occurred with the appearance of the bow-and-arrow [15], which would be dated at Sibudu to around 64 ka BP.

In this paper, we focus on an unpublished collection of bifacial serrated points that was discovered in the deep deposits of the site of Sibudu; these ancient occupations were individualized under the name of the “serrates layers”. These serrated pieces, also mentioned for the neighboring site of Umhlatuzana [7,16], derive from a well-controlled sedimentary context that precedes 77 ka BP. The results of an integrated study tackling the question of the manufacturing techniques and the use-mode of bifacial serrated points at Sibudu are presented here. Our technological and functional study provides significant new information about Sibudu and the South African MSA: 1) it reinforces and pushes back in time the evidence for the use of the pressure technique during the MIS5 in South Africa; 2) it enriches our understanding about projectile points in the MSA; 3) it contributes to current debates about the nature and diversity of bifacial expression in the MSA; and 4) it provides valuable new information for reflecting on the significance of serrated elements in the history of techniques in general, and for the South African MSA in particular.

## Site context and stratigraphy

**Fig 1:**
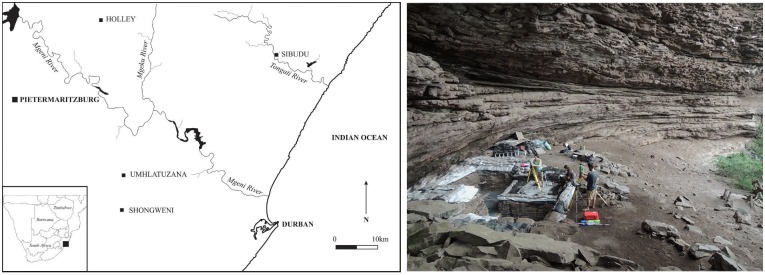
Sibudu Cave. Location of Sibudu in KwaZulu-Natal (left; after [21]); view on the excavation area (right) (The individuals in this picture have given their written informed consent).

Sibudu Cave, is situated approximately 40 km north of Durban and 15 km inland of the Indian Ocean. It is a rock shelter perched on a steep west-south-west facing cliff 20 m above the uThongathi River in the northern part of KwaZulu-Natal Province (KZN), South Africa (Fig 1). During a marine regression, the riverbed cut into the Natal Group sandstone cliff, which led to the formation of the shelter. The bedrock and sediments of the shelter slope steeply from north to south. The excavation area is located at an altitude of approximately 100 m above mean sea-level in the northern part of the shelter, where the deposits are thickest [17–19]. Paleoenvironmental reconstruction from sedge seeds and faunal remains indicate a mosaic of habitats during all occupation layers of Sibudu [20].

**Fig 2:**
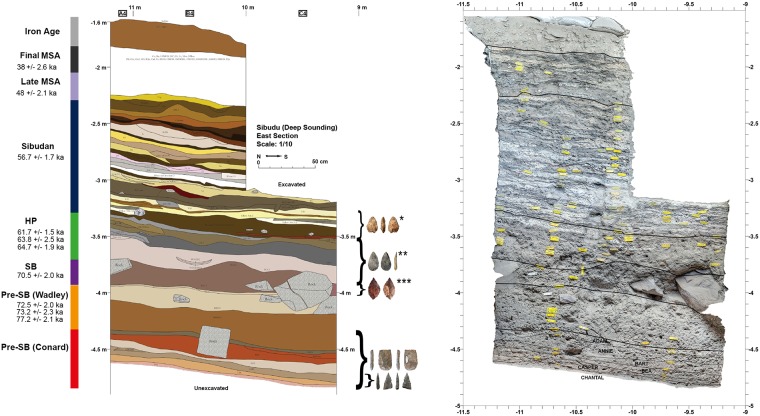
Stratigraphic main section of the deep sounding (east profile) of Sibudu including ages of the archaeological phases and indication of the presence of bifacial or serrated pieces. (* lower Sibudan (Will and Conard 2016), ** Howiesons Poort (de la Peña et al. 2013), ***Still Bay (Soriano et al. 2015) (left); Orthophotography of the east profile (right).

Unlike the upper part of the deposits that are predominantly composed of anthropogenic deposits [19,22], the stratigraphy recently exposed from the lower part of the deposits consists of fairly homogeneous ashy, sandy silts with major phases of rock fall. The archaeological layers are very rich and composed of stone and bone artefacts together with botanical remains. Mineralogical studies conducted by Christopher Miller and Susan Mentzer (University of Tübingen) identified important diagenetic processes [23,24]. In general, the sedimentary deposits show no significant reworking or mixing [21,22]. The over 3 m deep complete cultural sequence mainly comprises MSA layers spanning a time range of older than 77 to 37 ka BP (Fig 2). Several OSL and radiocarbon dates have been obtained from the MSA deposits [17,18,25,26] and new luminescence dates are in progress by Chantal Tribolo (CNRS, University of Bordeaux-III) [23].

The sequence includes occupations that are characteristic for the final MSA, late MSA, post HP, HP and SB. The lowermost layers excavated by Wadley (Light Brownish Grey (LBG) and Brown Sand (BS), before a team directed by Conard took the head of the excavation, were informally named ‘pre-SB’ (The field permit was issued by AMAFA, Heritage KwaZulu-Natal number 2931CA070, reference 0011/14). The pre-SB excavated by Wadley, including LBG and the upper part of BS (BS to BS10), describe lithic assemblages with flakes as well as blades and few formal tools, excluding bifacial pieces with the exception of one specimen from LBG; though this probably derived from the directly overlying SB layers [2,27]. The younger ‘pre-SB’ strata LBG yielded two OSL ages of 73.2 ±2.3 and 72.5 ±2.0 ka BP, while the layer BS yielded an OSL age of 77.3 ±2.7 ka BP [25]. In the new excavations, new layers below BS were identified (from the youngest to the oldest): Burnt Mouse (BMo), Red-Brown Silt (RBS), Grey Sandy Silt (GSS), Gray-Brown Patchy (GBP), Light Brown Compact (LBC), Pink Sandy Silt (PSS), Grey Mouse (GMo) and Compacted Orange Patchy (COP). In addition to these descriptive field designations, we assigned names of people starting from top to bottom with Adam, Annie, Bart, Bea, Casper, Chantal, Danny and Darya modified from systems used by Parkington and colleagues on the Western Cape. These are the layers that are under consideration in the present study.

## The lithic assemblages under consideration

This study primarily includes the lithic material of the basal layers Adam, Annie, Bart, Bea, Casper and Chantal from squares A4, B4, C4, A5, B5 and C5 (Table 1). However, the analyses also include the typological corpus of the lowermost layers Danny and Darya, as we identified similar characteristics and consider they belong to the same technological tradition. The lithic artefacts correspond to an area of approximately 4 m^2^ (ca. 1 m^3^ of sediment) excavated in five field seasons from 2012–2016. Due to the high find density of lithic single finds (Table 1), we used a cut off size of 30 mm for the blanks, while cores, core fragments, tools and tool fragments were recorded regardless of their size. The high ratio of small debitage products to single finds of 90:10% indicates intense on-site stone knapping activities with little post-depositional disturbance or sorting based on size. The typological classification complied with the commonly used terminology for the southern African MSA [28–30]. However, we also classified specific tools according to the recently defined terms based mainly on techno-functional analysis, including Tongatis, naturally backed tools (NBTs) and asymmetric convergent tools (ACTs) (see for definitions [31,32]).

**Table 1:** Sibudu Cave, all basal layers.

Layer	Field Description	Single finds (>30 mm)	Small debitage (<30 mm)	Total lithics	Sediment volume (m^3^)	Find density (n/m^3^)
**ADAM**	BMo	1814	15245	17059	0.122	139827.9
**ANNIE**	RBS	1045	9089	10134	0.092	110152.2
**BART**	GSS	1165	10545	11710	0.183	63989.1
**BEA**	GBP	1625	16524	18149	0.229	79253.3
**CASPER**	LBC	1434	16462	17896	0.195	91774.4
**CHANTAL**	PSS	1120	8937	10057	0.158	63651.9
**Total**		**8203**	**76802**	**85005**	**0,979**	**86828.4**

Frequency of lithic single finds and small debitage products, sediment volumes and find density.

Different raw materials are represented in the lithic assemblages. They are dominated by dolerite, followed by quartzite, sandstone, hornfels and quartz (Table 2). Little variation is noticed between the layers under study. All raw materials are of local origin (<5 km), except the hornfels that likely originates from Black Mhlasini River from a semi-local perimeter (< 20 km) [33].

**Table 2:** Sibudu Cave, basal layers under study.

Layer	Dolerite		Hornfels		Quartz		Quartzite		Sandstone		Other		Total	
	(N)	%	(N)	%	(N)	%	(N)	%	(N)	%	(N)	%	(N)	%
**ADAM**	1580	87.1%	37	2.0%	33	1.8%	97	5.3%	61	3.4%	6	0.3%	**1814**	**100%**
**ANNIE**	851	81.4%	31	3.0%	10	1.0%	74	7.1%	79	7.6%	-	0.0%	**1045**	**100%**
**BART**	938	80.5%	22	1.9%	49	4.2%	64	5.5%	88	7.6%	4	0.3%	**1165**	**100%**
**BEA**	1303	80.2%	41	2.5%	45	2.8%	76	4.7%	151	9.3%	9	0.6%	**1625**	**100%**
**CASPER**	1087	75.8%	30	2.1%	62	4.3%	87	6.1%	161	11.2%	7	0.5%	**1434**	**100%**
**CHANTAL**	882	78.8%	13	1.2%	19	1.7%	88	7.9%	115	10.3%	3	0.3%	**1120**	**100%**
**Total**	**6641**	**81.0%**	**174**	**2.1%**	**218**	**2.7%**	**486**	**5.9%**	**655**	**8.0%**	**29**	**0.4%**	**8203**	**100%**

Frequency of raw materials.

**Fig 3:**
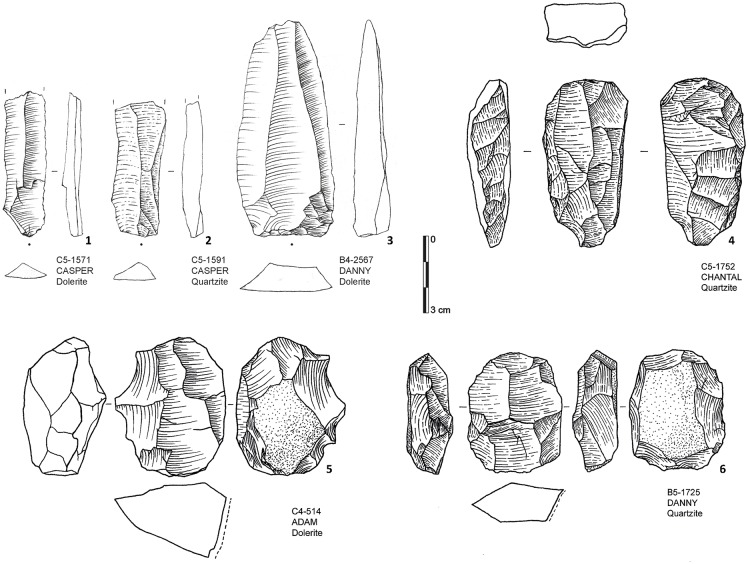
Sibudu basal deposits—Assemblage composition. Blades (1–3); Laminar cores (4–6).

Our preliminary study emphasizes the presence of one main laminar reduction sequence (Fig 3; Table 3). In parallel, we notice the presence of a bifacial technology that is based on the shaping of large blanks. We include all tools either bifacially retouched or bifacially shaped (for definition see [34]) in our bifacial category. These bifacial pieces are made from different raw materials, but preferentially from dolerite, quartz and quartzite (Table 4). They represent in total 21.4% of the formal tools, which also include various forms of unifacial points, scrapers and denticulates (Table 5).

**Table 3:** Sibudu Cave, all basal layers.

Layer	Blanks		Tools		Cores		Hammerstones		Debris		Total	
	(N)	%	(N)	%	(N)	%	(N)	%	(N)	%	(N)	%
**ADAM**	1630	89.9%	124	6.8%	22	1.2%	10	0.6%	28	1.5%	**1814**	**100%**
**ANNIE**	947	90.6%	68	6.5%	15	1.4%	5	0.5%	10	1.0%	**1045**	**100%**
**BART**	1050	90.1%	80	6.9%	16	1.4%	8	0.7%	11	0.9%	**1165**	**100%**
**BEA**	1463	90.0%	146	9.0%	6	0.4%	-	0.0%	10	0.6%	**1625**	**100%**
**CASPER**	1302	90.8%	111	7.7%	6	0.4%	1	0.1%	14	1.0%	**1434**	**100%**
**CHANTAL**	1015	90.6%	86	7.7%	6	0.5%	4	0.4%	9	0.8%	**1120**	**100%**
**Total**	**7407**	**90.3%**	**615**	**7.5%**	**71**	**0.9%**	**28**	**0.3%**	**82**	**1.0%**	**8203**	**100%**

General technological classification of lithic artefacts.

**Table 4:** Sibudu, basal layers under study.

Layer	Field Description	Dolerite		Hornfels		Quartz		Quartzite		Sandstone		Chert		Total	
		(N)	%	(N)	%	(N)	%	(N)	%	(N)	%	(N)	%	(N)	%
**ADAM**	BMo	4	33.3%	1	8.3%	6	50.0%	1	8.3%	-	0.0%	-	0.0%	**12**	**100%**
**ANNIE**	RBS	6	50.0%	1	8.3%	1	8.3%	4	33.3%	-	0.0%	-	0.0%	**12**	**100%**
**BART**	GSS	8	32.0%	1	4.0%	14	56.0%	1	4.0%	1	4.0%	-	0.0%	**25**	**100%**
**BEA**	GBP	13	31.7%	4	9.8%	13	31.7%	8	19.5%	-	0.0%	3	7.3%	**41**	**100%**
**CASPER**	LBC	12	40.0%	2	6.7%	12	40.0%	4	13.3%	-	0.0%	-	0.0%	**30**	**100%**
**CHANTAL**	PSS	8	44.4%	1	5.6%	3	16.7%	6	33.3%	-	0.0%	-	0.0%	**18**	**100%**
**DANNY**	GMo	6	31.6%	-	0.0%	2	10.5%	11	57.9%	-	0.0%	-	0.0%	**19**	**100%**
**DARYA**	COP	5	38.5%	-	0.0%	1	7.7%	7	53.8%	-	0.0%	-	0.0%	**13**	**100%**
**Total**		**62**	**36.5%**	**10**	**5.9%**	**52**	**30.6%**	**42**	**24.7%**	**1**	**0.6%**	**3**	**1.8%**	**170**	**100%**

Frequency of raw material types in bifacial pieces.

**Table 5:** Sibudu, basal layers under study.

Layer	Bifacial Tool*		Pointed Form**		Scraper-like Form***		Denticulate		Indet. Fragment		Total	
	(N)	%	(N)	%	(N)	%	(N)	%	(N)	%	(N)	%
**ADAM**	12	9.7%	66	53.2%	19	15.3%	8	6.5%	19	15.3%	**124**	**100%**
**ANNIE**	12	17.6%	31	45.6%	14	20.6%	4	5.9%	7	10.3%	**68**	**100%**
**BART**	25	31.3%	32	40.0%	8	10.0%	4	5.0%	11	13.8%	**80**	**100%**
**BEA**	41	28.1%	62	42.5%	18	12.3%	7	4.8%	18	12.3%	**146**	**100%**
**CASPER**	30	27.0%	50	45.0%	13	11.7%	10	9.0%	8	7.2%	**111**	**100%**
**CHANTAL**	18	20.9%	39	45.3%	17	19.8%	2	2.3%	10	11.6%	**86**	**100%**
**DANNY**	19	17.6%	46	42.6%	31	28.7%	1	0.9%	11	10.2%	**108**	**100%**
**DARYA**	13	17.8%	25	34.2%	20	27.4%	1	1.4%	14	19.2%	**73**	**100%**
**Total**	**170**	**21.4%**	**351**	**44.1%**	**140**	**17.6%**	**37**	**4.6%**	**98**	**12.3%**	**796**	**100%**

Frequency of tool types:

*Including all serrated pieces;

**Including ACTs and Tongatis;

***Including NBTs).

One striking element within the bifacial component is the presence of serrated pieces (for a definition see [16,35,36]). We acknowledge that there is variability in the morphometric attributes of what has been defined as serrated points. Akerman and Bindon [36] for example establish a distinction between the dentate, the denticulate and the serrated points on the basis of the size of the notches and their separation. In the following study, however, we use the word “serration” as a generic term encompassing all pieces with one or two edges that have been regularly shaped with contiguous notches creating fine triangular teeth, regardless of their dimensions and morphologies.

## Material under study and research questions

**Fig 4:**
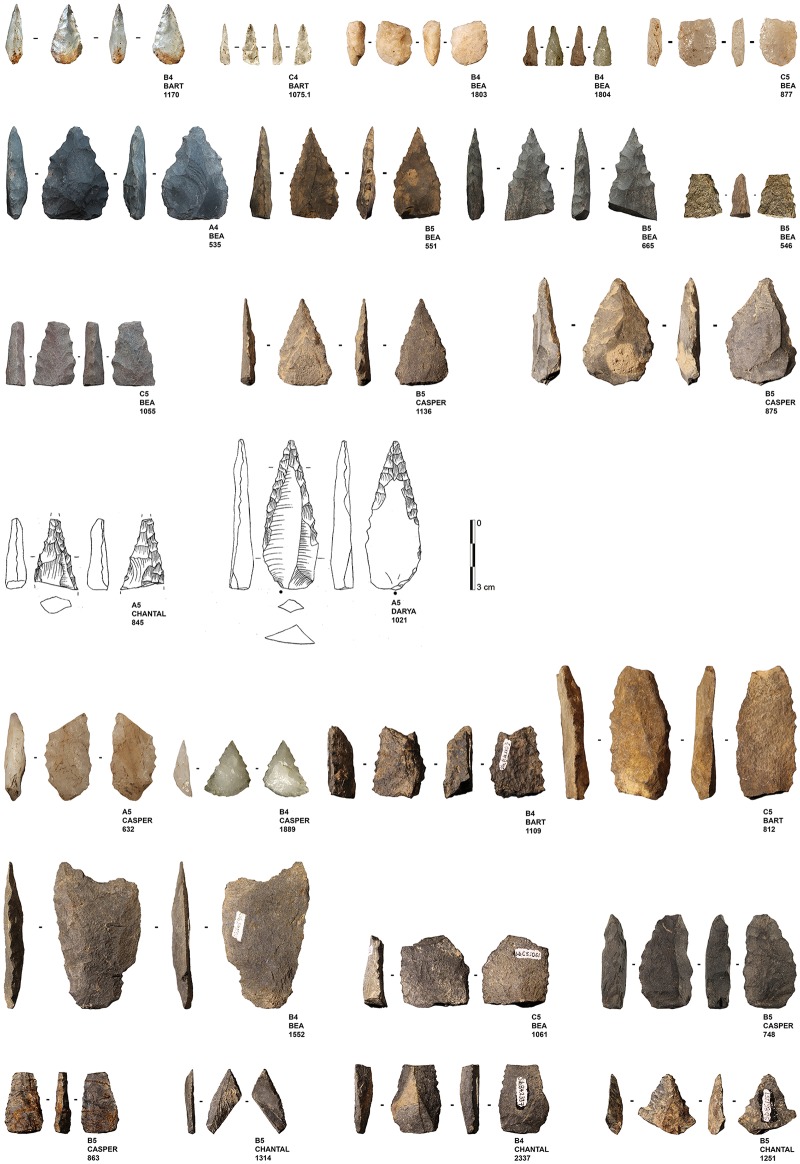
Sibudu basal deposits—Overview of all serrated pieces. Analysed serrated pieces (rows 1–4); Unused and unanalysed serrated pieces (rows 5–7).

The serrated pieces come from the layers Bart, Bea, Casper, Chantal and Darya. We identified a total of 25 serrated pieces (Fig 4) (All tools are under storage at the museum of Pietermaritzburg). For the present study, we assess the technological and functional variability of this assemblage.

**Fig 5:**
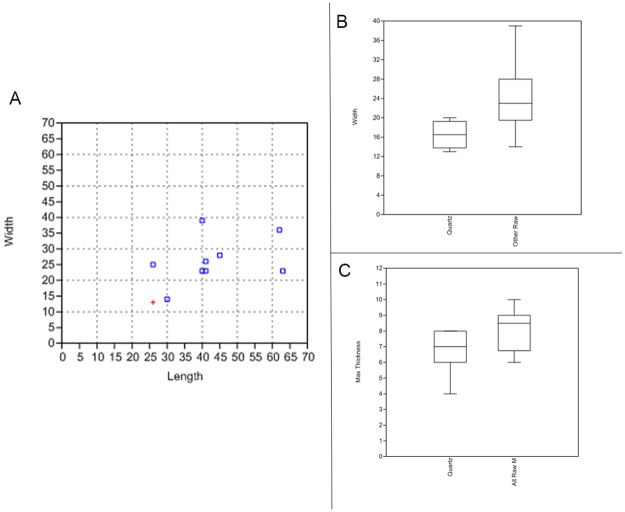
Sibudu basal deposits. **A**—scatter plot of widths and lengths of all complete serrated pieces (red cross–quartz, blue squares–dolerite and hornfels); **B**—Box plot of maximal width of all serrated pieces in comparison to the used points exclusively; **C**—Box plot of maximal thickness of all serrated pieces in comparison to the used points exclusively.

The pieces have a mean length of 41 mm, a mean width of 23 mm and a mean thickness of 8 mm (Fig 5; Table 6). Their length-width-ratio is 1.73 +/-0.5 and their width-thickness-ratio is of 2.9 +/-0.8. The population under study has a bilateral symmetry and a general triangular morphology. The profile of the tools is straight but the delineation of their edges is variable, often sinuous in the proximal part and more regular in the distal part. This regularity partly relates to the alternation of the notches when they are bifacial, either the notches are bifacially symmetric (the bifacial notches originate in a same contact point) or asymmetric (the bifacial notches originate next to each other’s contact point). We found some variability on the cross-sections which range from being bevelled, biconvex or plano-convex, suggesting a degree of variability in the bifacial reduction sequences.

**Table 6:** Sibudu, basal layers under study.

Layer	Square	ID	Raw Material	State	Max Length	Max Width	Max Thickness	W at base	Average serration width
BART	B4	1109	Dolerite	Basal Fragment	32	23	13	22	3
	B4	1170	Quartz	Complete	26	13	7	9	2
	C4	1075–1	Quartz	Distal Fragment	17	7	4	-	2
	C5	812	Dolerite	Basal Fragment	56	28	11		4
BEA	A4	535	Dolerite	Complete	40	39	9	24	5
	B4	1804	Quartz	Distal Fragment	16	7	6	-	1
	B4	1803	Quartz	Basal Fragment	18	16	8	12	3
	B4	1552	Dolerite	Complete	62	36	8	16	6
	B5	551	Hornfels	Complete	41	23	9	18	2
	B5	665	Dolerite	Proximal Fracture	40	21	7	-	4
	B5	546	Dolerite	Medial Fragment	19	16	6	-	4
	C5	877	Quartz	Basal Fragment	21	17	6	11	3
	C5	1055	Quartzite	Medial Fragment	28	18	8	-	5
	C5	1061	Dolerite	Basal Fragment	34	31	10	27	3
CASPER	B4	1889	Quartz	Distal Fragment	24	19	8	-	3
	B5	1136	Dolerite	Complete	41	26	6	21	3
	B5	875	Hornfels	Complete	45	28	9	22	5
	B5	748	Dolerite	Complete	40	23	10	16	4
	A5	632	Quartz	Basal Fragment	38	20	8	13	5
	B5	863	Dolerite	Basal Fragment	28	17	7	15	2
CHANTAL	B5	1314	Dolerite	Distal Fragment	30	14	3	-	1
	B4	2337	Dolerite	Basal Fragment	30	22	8	17	3
	B5	1251	Dolerite	Distal Fragment	26	25	7	-	4
	A5	845	Dolerite	Distal Fragment	30	18	8	-	4
DARYA	A5	1021	Dolerite	Complete	63	23	9	-	4

Description of serrated pieces (including specimen numbers).

The population of serrated pieces shows some variability that interestingly, relates to the nature of the rocks that have been shaped. In this regard, pieces made from quartz can be distinguished from those made from quartzite, hornfels and dolerite. Firstly, there are differences in tool size, with quartz pieces being smaller than those manufactured from other raw materials (Figs 4 and 5). In addition, there is some variation in the morphologies of the bifacial pieces: when bases are preserved, they are semi-circular in the case of quartz and straight for the other raw materials.

To interpret size and shape differences, we first have to acknowledge the technological variability within this population or, in other words, their *chaînes opératoires*. Although our sample is small, limited to 25 pieces, and we presently lack a global understanding of the associated lithic assemblages, we can nevertheless present some preliminary observations based on our examination. First of all, the sample can be subdivided into two groups: bifacially shaped blanks that were bilaterally serrated (n = 11) and bifacially (or unifacially) retouched blanks that were laterally or bilaterally serrated (n = 14). While we recognize two techno-typological groups, we cannot discern clear differences between them in terms of their morphology or dimension. In general, we suggest that these two reduction sequences reflect different ways to obtain a similar result, namely a triangular elongated tool with serrated edges. However, we again observe a difference between the quartz pieces and those made from other rock types, as the quartz pieces always correspond to the category of bifacially shaped blanks that have been bifacially serrated. We will further elaborate on how we interpret differences between the raw materials that were used by inhabitants of Sibudu, in the discussion.

For a better understanding of the *chaînes opératoires*, we integrate some preliminary observations based on the bifacial pieces (not serrated) derived from the same layers as the serrated pieces. We will refer to these non-serrated pieces as the ‘formal bifacial pieces’. Of the total number of 168 bifacials, 143 are formal bifacial pieces, and the rest are serrated. This means that approximately one out of every six bifacial pieces was serrated.

The *chaîne opératoire* as deduced from the full assemblage of bifacial pieces begins with the knapping of elongated products. These blanks indicate that there would not have been a great degree of pre-determination and from the presence of cortex often seem to originate from an initial phase of the core reduction. Currently, there appears to be no specific core reduction sequence that was oriented toward the production of blanks to be selected for bifacial shaping. Once the toolmakers selected the appropriate piece, they shaped it by direct percussion until they achieved the desired dimension and shape in terms of bilateral and bifacial symmetries. The set-up of the desired forms was therefore related to the different parameters of the percussive technique, i.e. the force that was applied, the motion of the percussion (more or less tangential) and the location of the impact point (more or less at the interior of the edge). It is only after this shaping procedure that a series of notches was set up, intended to serrate the edges.

Interestingly, no firm signs of reworking were recognised on the serrated pieces, suggesting that the serration was not only the last stage of the manufacturing process, but also its final stage. It suggests to us the existence of a techno-economical distinction between the serrated bifacial pieces that represent finished tools (in terms of morphometric characteristics) and the formal bifacial pieces that seem to have been shaped at different times throughout their life cycles. We can draw two primary conclusions from this observation. First, the serrated pieces under study can all be regarded as finished forms, with the exception of the pieces that fractured during the manufacturing process. Second, the variability of raw materials, dimensions and shapes is not related to differences in their stages of discard.

We note a certain amount of variation between the serrated points according to the notches. First of all, we have to acknowledge that the serration is not equally explicit on all the pieces. Some tools are regularly serrated all along their edges while others show more discontinuous serrations. Some tools have deep notches while on others they are very shallow. Also, some tools are bifacially notched while others are only unifacially serrated. The serration never occurs at the base of the points—notches are always visible on the medial part and, to a lesser extent, on the distal part. We consider that this variability is related to the function of the pieces or to the tool that was used to serrate the blank. To help determine the causes of variability we took measurements of all notches (n = 288, mean of 11 to 12 notches measured per serrated piece), using Photoshop software. The notches have a mean width of 3.2 mm, with a standard deviation of 1.7 mm, and a mean depth of 0.5 mm, with a standard deviation of 0.3 mm.

Apart from the raw material, functional factors may explain the variation we observe among the serrated pieces. For the functional analysis, 18 bifacially serrated points or point fragments were considered, as the rest of seven pieces appear not to have been completed, and thus, were never used. The pieces come from the layers Bart, Bea, Casper and Chantal, and also include one serrated piece from the lowermost layer Darya. From the total sample of 18 bifacial serrated points and point fragments, one serrated point with a broken tip (B5-863) had to be excluded due to significant iron deposits and cracking, which prevented a reliable functional analysis. An independent use-wear and residue analysis were performed on the remaining 17 artefacts (Table 7). Different microscopic techniques were combined and the analysis was performed in specific stages: (1) a broad screening performed by Veerle Rots to understand general wear patterns and to allow an informed selection of tools that would be relevant for residue analysis, (2) residue analysis of the stone tools performed by Carol Lentfer, (3) use-wear analysis of the stone tools performed by Veerle Rots.

**Table 7:** Analytical protocol as applied to the serrated points.

Layer	Unit	ID	Raw material	Complete / Fracture	Analysed sediment sample	Residues					Wear Traces	
						in-situ analysis	localised pipette extractions	USB extraction	analysis of extracted residues	SEM	low magnification analysis	high magnification analysis
GSS	B4	1170	crystal quartz	complete	-	x	5	D,P-M	x	-	x	x
GSS	C4	1075.1	crystal quartz	distal fragment	-	x	2	D,D-M	x	-	x	x
GBP	B5	665	dolerite	nearly complete (small proximal fracture)	-	x	3	-	x	x	x	-
GBP	B5	551	hornfels	complete	x	x	3	-	x	x	x	x
GBP	B4	1804	milky quartz	distal fragment	-	x	3	-	x	x	x	x
GBP	C5	877	milky quartz	basal fragment	-	x	4	M,P	x	-	x	x
GBP	B4	1803	milky quartz	basal fragment	-	x	1	M-P	x	-	x	x
GBP	A4	535	dolerite	complete	-	x	3	-	x	x	x	x
GBP	B5	546	dolerite	medial fragment	-	x	2	M	x	-	x	x
GBP	C5	1055	quartzite	medial fragment	-	-	-	-	-	-	x	x
LBC	B5	1136	dolerite	complete	x	x	1	D,P, P-M	x	-	x	x
LBC	B5	875	hornfels	complete	x	x	4	D,P, P-M	x	x	x	x
LBC	B4	1889	milky quartz	distal fragment	-	x	3	D-M	x	-	x	x
LBC	A5	632	milky quartz	basal fragment	x	x	3	M,P	x	-	x	x
LBC	B5	748	dolerite	complete	-	-	-	-	-	-	x	-
PSS	A5	845	dolerite	distal (medial) fragment	-	x	3	D,M	x	-	x	x
COP	A5	1021	dolerite	complete	x	x	2	D,D-M	x	-	x	x

(D = distal, M = medial, P = proximal)

The serrated pieces from Sibudu represent one peculiar innovation that has presently received little attention. While serration has long been associated with the pressure technique (Crabtree 1973), the question of the technique used to notch the edges of the bifacial blanks still needs to be firmly demonstrated. Similarly, the functional reason for serrated edges still remains to be clarified. It was with two questions in mind—1) how was the serration applied?, 2) what was the serration for?–that we established our methodology.

## Methods of the functional analysis

Tools were first screened under a binocular stereoscopic microscope (magnifications up to x56). Potential evidence of use and hafting was documented and a working hypothesis was proposed. Based on this broad examination, relevant tools were selected for detailed functional analysis including residues and use-wear (Table 7). While a general priority listing of the tools was made, no details of the screening were communicated to the residue analyst.

**Fig 6:**
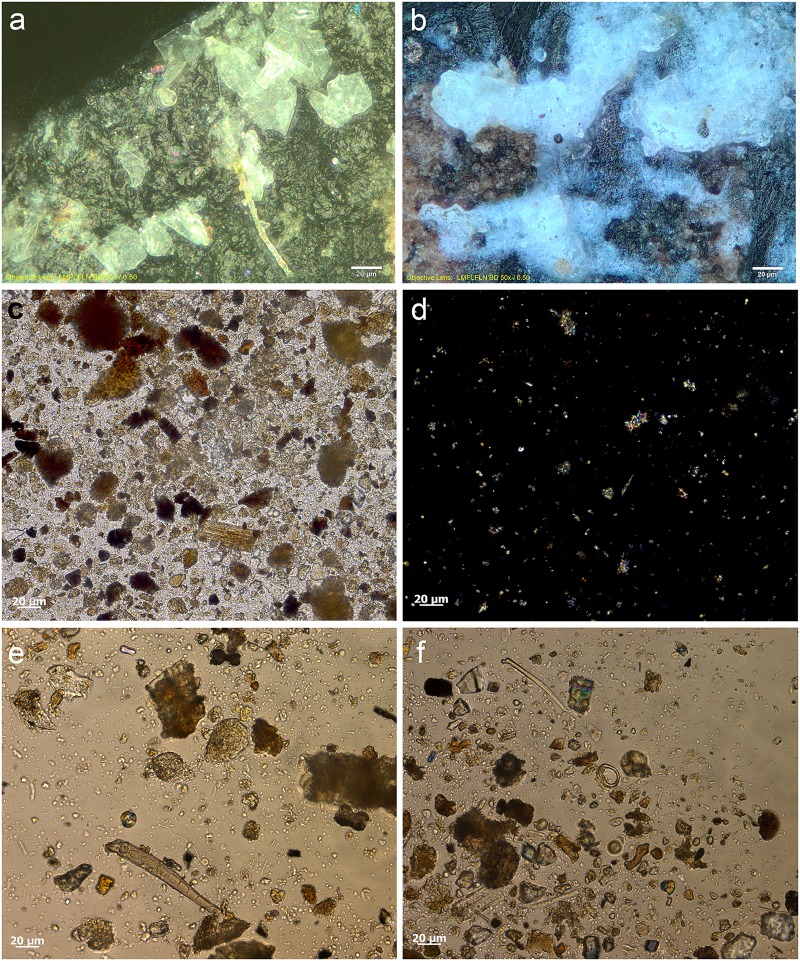
Examples of contamination and residues from associated sediment. **a)** abundant skin tissue from post excavation handling on the ventral left proximal edge of 665 (x500, bright field); **b)** plasticine contamination on the dorsal left proximal end of 1170 (x500, dark field); **c)** elongate phytoliths, articulated plant tissue cf. Commelinidae, charred palynofacies and other sedimentary particles in sediment sample from layer Bea (x400, transmitted light); **d)** pleochroic particles of CaCO3 from sediment sample from layer Casper (x400, transmitted polarised light); **e)** spores, sclereid and lobed CaCO3 crystal from hyrax faecal sample (the crystal is typically found in urine) (x400, transmitted light); **f)** other pleochroic crystals, spores and fibre from hyrax faecal sample (x400, transmitted light).

Most tools prior to screening were unwashed and unhandled. However, all had been handled for photography prior to the detailed functional analysis. Such handling resulted in contamination residues, consisting of skin flakes from dust and handling, fibres including easily recognised synthetic fibres, plasticine that was used for mounting some specimens while photographing them, and possibly some of the starch granules (Fig 6a and 6b). To avoid further chances of contamination starch-free gloves were worn during the screening process and subsequent residue analyses.

### Residue analysis of tools

Fifteen tools, including broken fragments, with diagnostic production and/or use-related attributes were selected for residue analysis. The analysis was based primarily on morphological identification of microscopic residues using stereo binocular, metallurgical, transmitted and occasionally scanning electron microscopy.

The initial stages of the analysis followed standard micro-residue analytical procedures (e.g., [37,38]) using an Olympus SZX7 binocular stereomicroscope and a Zeiss AxioZoom V16 zoom microscope to scan all surfaces of tools and record the distribution of discernible residues and sediment onto line drawings. Magnification ranges at this stage were from x5.6 to x180 and images were taken with the Zeiss V16 zoom microscope of selected residues. Following this initial procedure, tools were systematically examined at higher magnifications ranging from x50 to x1000 using Zeiss and Olympus metallurgical microscopes fitted with dark field and bright field incident light sources, polarising filters, differential interference contrast (DIC) and cameras. Locations of micro-residues were recorded onto line drawings and representative residues were photographed.

In a subsequent stage, following procedures adopted by Loy [39] and others (e.g., [40]), transmitted light microscopy was employed to confirm presence and identification of residues observed with reflected light. This procedure also enables detection of other diagnostic residues such as small fibres, starch, phytoliths, other plant and animal tissue fragments, crystals and minerals that may not, or could not have been detected with reflected light. For each tool, various locations noted to have residues and/or locations that would be expected to have diagnostic residues if the tool had been used were selected for sampling. A small amount of distilled water (a minimum of 200 μl) was pipetted onto each of these locations and the wetted sample residue was redrawn into the pipette then transferred to a microscope slide. Dependent upon the adherence of residues, additional application of water and/or abrasion with the pipette tip to help remove particles was sometimes utilised. Slide samples were mounted in distilled water and examined under x100 to x400 magnification using a Zeiss Axioscope.A1 microscope with normal and polarised light as well as DIC. Presence of individual types of residue was recorded and relative abundance of significant residues was noted. Images were taken with a Zeiss AxioCam ICc5 microscope dedicated camera.

To further assess residue presence and abundance, tools were placed into an ultra-sonic bath (USB) with distilled water to remove samples of remaining residues from tool surfaces. To help remove contaminants such as fibres and skin cells derived from handling and exposure to air-borne dust, tools were lightly washed in distilled water prior to USB. Treatment was five minutes per bath. Distal and proximal portions of unbroken tools were treated separately. Likewise, for broken pieces, distal or proximal and medial sections of tools were treated separately. For whole tools medial sections were sampled by immersing the already treated proximal ends together with un-treated medial sections into water. Residues removed in this way were analysed in accordance with the pipette samples. In instances where very large amounts of residue were obtained via the USB, sub-samples were scanned until the number of new types encountered in samples plateaued. It should be noted that four tools with sufficient residues for gas chromatography-mass spectrometry (GC-MS and/or GC-GC) analysis and the proximal to medial portion of another tool were not given the USB treatment. The inclusion of the USB procedure has the advantage that samples of all relevant residues were extracted and no further precautions were necessary during subsequent handling and analysis.

Selected residues on five tools (four selected for GC-MS) were examined and characterised with the environmental SEM facility (FEI XL30 ESEM-FEG) available at the University of Liège. Micrographs were obtained for respective residues and elemental energy dispersive X-ray (EDX) analysis was undertaken. No coatings were applied during this procedure.

### The sedimentary context: Implications for preservation of residues and background ‘noise’

To facilitate our interpretations of production/use-related residues vs environmental/post-depositional residues, analysis of 7 sub-samples of sedimentary layers associated with the stone tools was undertaken (Table 8). To ensure homogeneity of the sediment samples, collection bags containing samples were well-shaken prior to treatment. One cm^3^ of each sample was placed into a 6 ml vial with distilled water and disaggregated by vigorous shaking for 2 minutes, then USB treatment for five minutes. Sub-samples of the disaggregated samples were transferred to microscope slides and analysed in accordance with the procedures used for the pipette and USB samples of tool residues.

**Table 8:** Summary of residues found in sediment samples.

Code (associated tool)	Animal		Plant			Mineral				Other		Comments
	Tissue	Bone	Tissue	Fibre	Phytolith	Vivianite	Hematite	Crystal	Sedimentary particles (indet.)	Micro-charcoal/ TA palynofacies	Tissue (indet.)	
**GSS (650)**			ab		ab				ab	ab	+	Commelinidae tissue and phytoliths abundant—diagnostic sedge tissue present. Charred particles abundant.
**LBC (2195)**			c		c				ab	ab	+	Commelinidae tissue and phytoliths present. Diagnostic morphotypes absent.
**LBC (632)**			vc		c	?			ab	vc	+	Commelinidae tissue and phytoliths present. Nodular globular phytoliths cf. Dilleniidae present. Aggregates of pleochroic CaCO3 particles very common.
**LBC (1136)**			vc		c			CaCO3	ab	ab	+	Commelinidae tissue and phytoliths present. Nodular globular phytoliths cf. Dilleniidae present.
**GBP (551)**			ab	+	ab				ab	ab	+	Commelinidae tissue and phytoliths present including grass trichomes. Nodular globular phytoliths cf. Dilleniidae present.
**GBP (1006)**		?	ab	+	ab		+	CaCO3	ab	ab	+	Commelinidae tissue and phytoliths from grass and sedge present. Nodular globular phytoliths cf. Dilleniidae present.
**COP (1021)**	+		ab	+	ab				an	ab	+	Commelinidae tissue and phytoliths mostly from grass.

+ (present), c (common), vc (very common), ab (abundant).? means uncertain identification.

Starch, animal tissue traces and vivianite, a hydrated iron phosphate mineral often found in association with animal residues, were extremely rare in the sediment samples. Therefore, their presence on tool surfaces can be due to more favourable micro-environmental conditions enabling their preservation or to being used in tool manufacture and/or related to tool function. The preservation of a range of other organic residues in the sediment samples was excellent (Table 8) and provides a background by which to assess ‘noise’ residue, unrelated to tool production and function. Charred plant fragments and phytoliths indicated an abundance of burnt *Commelinideae* (grass/sedge) plant material and some woody angiosperm plants including *Dilleniidae* species (e.g., *Moraceae* and *Euphorbiaceae* spp.) (Table 8; Fig 6c and 6d). Diagnostic epidermal long cell and short cell phytoliths from panicoid grasses were occasionally present, but absent and/or rare in most sediment samples. Hence, it is likely that the *Commelinidae* micro-fossils were primarily derived from sedges. This is in accordance with previous studies of the higher stratigraphic levels that identified an abundance of burnt plant material, particularly sedge, interpreted as being from bedding material that was burnt frequently for site maintenance [41], and ultimately contributed to the creation of the distinct laminated strata of the MSA sedimentary sequence of Sibudu Cave, or so it is thought [19,22,42].

Plant remains would have been brought into the rock shelter by other means too, not only humans, and the taphonomic implications of this, especially with regard to preservation of residues and background ‘noise’ should be considered. At Sibudu, an obvious source would be the rock hyrax (*Procavia capensis*); currently a common inhabitant of the rock shelter and highly likely to have lived there during the MSA. This small-sized mammal browses upon a broad spectrum of plants, including sedges and grasses, as well as twigs, shoots, leaves and flowers from shrubs and small trees, lichens and liverworts [43]. Although they generally eat on the spot (i.e., close to the food source), and are not known for carrying food back to shelters for eating, they do have a peculiar habit of defecating under shelter [44]. By so-doing, they create large, often well-stratified urino-faecal middens, packed full of plant remains and very useful for paleoenvironmental analysis [43–45]. Currently, one such midden is on an elevated platform in the south-western section of the Sibudu rock shelter and an analysis of the masticated components of a sample of faecal pellets from here, did indeed confirm the varied diet of rock hyrax, at least in the present day environment around Sibudu. The sample analysed consisted of large stellate trichomes, other trichomes, large sheets of epidermis, sclereids and woody tissue fragments from dicotyledinous plants, as well as grass and sedge epidermis and phytoliths, pollen, lichen and fungal spores. There were also remains of invertebrate appendages, exoskeletons, including fragments of millipede exoskeleton, and scales from the wings of butterflies or moths, which may or may not have been intentionally consumed. Therefore, given the likelihood of the rock hyrax being an inhabitant of the rock shelter in the MSA, it would undoubtedly have made substantial contributions to the plant material found in sedimentary deposits. Moreover, and perhaps most importantly for sedimentary formation processes, the rock hyrax has a unique physiology giving it the ability to concentrate its urine to reduce water loss, and allowing it to successfully live in arid environments [46]. The dried, carbonate-rich urine (hyraceum) from hyrax, is also likely to be a major source of the calcium oxalate crystals, granular casts and pleochroic calcium carbonate crystals (Fig 6e and 6f), very common components of the sediment samples. Furthermore, since it caps faecal deposits much like the calcitic concretions that caps sedimentary deposits in limestone caves, its dissolution and re-precipitation within the rock shelter deposits may have been instrumental to the formation of their finely laminated strata and the outstanding preservation of organic material.

### Wear analysis

For the wear analysis, all 17 tools were examined under a binocular stereoscopic microscope (magnifications up to x56; Zeiss and Olympus), a zoom microscope (Zeiss V16, magnification up to x180), and a metallurgical reflected-light microscope equipped with bright field illumination, polarising filters and DIC (magnifications up to x1000) (Olympus BX51M) according to established procedures (cf. highlighted in [47]). Pictures were taken with adapted microscope cameras (Olympus SC100). Attention was devoted to all types of traces including scarring, polish, striations and rounding. Given that all residue extractions were performed before the detailed use-wear analysis started, tools could be handled and cleaned with alcohol or acetone during analysis without the risk of contaminating or removing residues. This cleaning procedure is essential in order to be able to correctly observe polish and striations. Special attention was also devoted to fractures and fracture patterns. Projectiles were interpreted on a combination of different wear traces in specific patterns and no interpretation relied on one wear type only (cf. [14]). For describing fractures, terminological ambiguities were avoided as much as possible (cf. [48]).

### Reference material

Interpretation of residues and wear traces were based on the experimental reference collection available at TraceoLab, University of Liège. This collection consists of modern comparative reference materials and over 3000 experimental tools produced in various ways and used in various activities both in the hand and hafted in various arrangements (e.g., [47]). The collection consists primarily of flint tools, but also tools made from chert, quartz, quartz crystal, obsidian, and dolerite. About 300 experimental flakes (used and unused) are currently available in raw materials that were used at Sibudu, especially dolerite. A specific experimental program on serrated pieces was initiated within the framework of this study (see below). Additionally, published and unpublished comparative reference databases were used for the residue analysis, including comprehensive starch plant fibre and phytolith modern comparative reference collections compiled by Lentfer (e.g., [49–51]). Also, with regard to iron oxide residue it should be noted that extreme care was taken for the identification of applied hematite on stone tool surfaces. Iron oxide occurs in dolerite and hornfels rock types (see [33]: Table 4a: 103) being often visible in the rock matrix in the form of olivine which weathers to goethite (hydrated iron oxide) and hematite (iron oxide). The latter is indicated by a distinct red colouration on weathered rock surfaces. Knapping experiments with dolerite found near the Sibudu rock shelter confirmed that the rock often broke along weathered fracture lines to reveal hematite on freshly broken surfaces. Hence, its classification as applied residue was dependent on its thickness and distribution and/or its association with other residues such as resin, fat, micro-charcoal, starch and other plant material or tissue used for compound adhesives or other hafting elements.

### Experimentation

A reference sample of serrated points was reproduced experimentally. Fourty-three serrated points were manufactured by a knapper with 20 years of experience, Christian Lepers (University of Liège): 21 out of quartz crystal, 8 out of quartz, 10 out of dolerite, and 4 out of flint. All blanks were shaped with a wooden hammer and serrated by pressure with a bone compressor. The goal of the knapper was to shape a pointed form with a maximal width located in the 1/3 proximal part, a bilateral symmetry and a point angle comprised between 45° and 55°. No special indications were given regarding the characteristics of the sections, nor of the bases. Once the blank was bifacially shaped, the instruction was to serrate the edges bifacially without any specification regarding the alternation of the notches.

Some points were not entirely finished due to them fracturing early in the production sequence (7 in crystal quartz, 4 in quartz, 3 in dolerite), or in a later production stage when they were close to being finished (2 in crystal quartz, 1 in dolerite). Three serrated points in crystal quartz were completed, but ended up very small owing to the correction of some fractures during the production process. All the points or preforms that fractured during production served as a comparative reference for the analysis of the archaeological points. Additionally, all the small shaping flakes were collected and in instances where no early fractures occurred, flakes were separated for each shaping phase (e.g., initial shaping, advanced shaping, finishing). These shaping flakes were examined, in particular their butt, to record the wear that results from the pressure flaking process.

**Fig 7:**
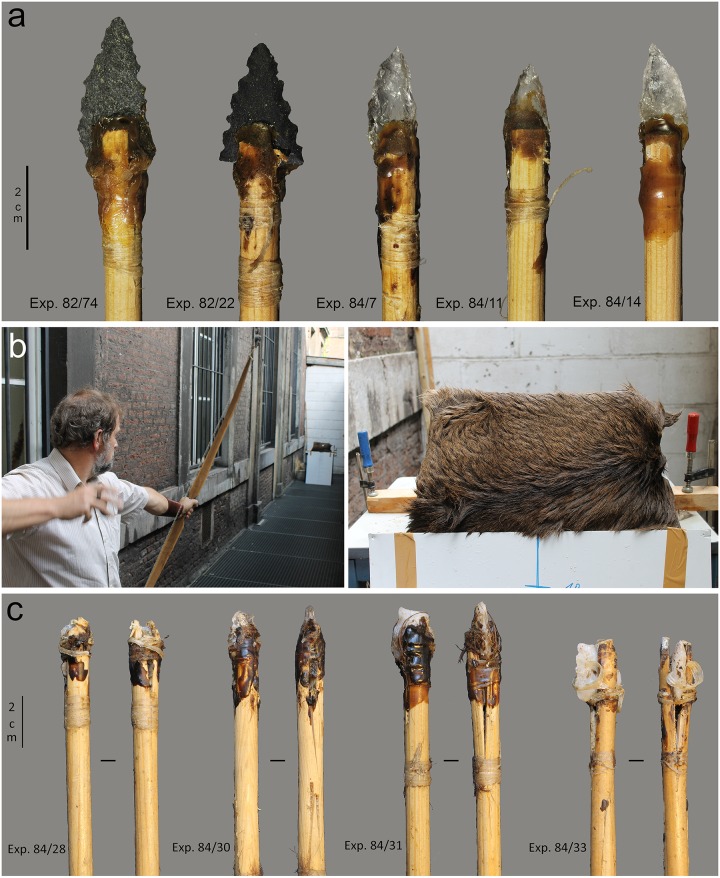
Projectile experiments. **a)** hafted experimental projectiles before the experiment; **b)** experimental set-up including target used on the right (The individual in this picture has given his written informed consent); **c)** hafted experimental projectiles after the experiment showing fractured projectile tips, cut bindings, as well as points that inserted into the shaft upon impact.

Twenty-three serrated points (9 in quartz crystal, 4 in quartz, 4 in flint, 6 in dolerite) were mounted in a split on the extremity of pine shafts (11/32 inch in diameter; average spin of 1.21 cm) with the aid of resin (70% natural pine resin, 30% beeswax) or resin combined with sinew bindings (Fig 7; Table 9). Points were shot into an artificial target with a 47 pound (draw weight) flat bow manufactured out of elm (30 inch draw distance) (Fig 7). Shots were taken at a distance of 10 m from the target. The target consisted of ribs incorporated in a ballistic gel (240–260 bloom; gelatine type A; 24h in cold chamber at 4°C; cf. [52]) and was covered with a remoistened animal hide (horse) fixed tightly around the gel. A double series of ribs was used to increase the chance of impact damage by contact against bone. The goal was not to produce an experimental set that would be representative in terms of fracture frequencies, but to supplement the existing projectile reference collection of the TraceoLab (nearly 500 points) with fracture points on samples of raw material and point morphology highly similar to the archaeological points. Points were shot once unless no damage occurred at all, in which case they were re-used. No point was shot more than four times and the total number of shots was 33 (Table 9). Two out of 23 points missed the target and hit a soft stone wall (itong), while 4 points successfully hit the target, but bounced off and hit the soft stone wall.

**Table 9:** Experimental data for the projectile experiments including all ballistic information.

Ref Nr	Raw material	Pine shaft morphology	Diameter of shaft	Gross mass of shaft (g)	Mass of stone point (g)	Mass hafted tool (g)	Type of fletching	surface de frottement empennage	Length of shaft (cm)	Total L shaft + point (cm)	Point of equilibrium of the finished tool (cm from the point tip)	FOC of the finished arrow (FOC ((L/2-Lgrav)/L)*100)	Fixation	Number of shots	Contact material	Penetration in target (cm)	Result
Exp. 82/22	dolerite	cylindrical	11/32	32.89	2.27	35.45	3 x 5 inch parabolic		82.0	84.0	41.2	0.95	glue	1	skin+gel	0	point breakage; dehafted
Exp. 82/74	dolerite			28.38	3.89	32.61			82.0	84.6	37.2	6.03	glue	1	wall	0	point breakage; dehafted
Exp. 82/135	dolerite			30.27	6.03	36.91			82.0	83.5	36.9	5.81	glue	2	(1) skin+gel; (2) skin+gel+wood	2.0	de-hafted
Exp. 82/250	dolerite			36.78		36.78			82.0	85.0	33.5	10.59	glue+sinew+glue	1	skin	0	de-hafted (shaft broke)
Exp. 82/251	dolerite			37,00		37			82.0	87.0	39.0	5.17	glue+sinew+glue	2	(1) skin+gel; (2) skin+gel+bone	(1) 9.5; (2) 5.5 + point	point breakage; dehafted
Exp. 82/252	dolerite			29.86		29.86			82.0	83.6	37.0	5.74	glue+sinew+glue	1	skin	0	de-hafted on impact
Exp. 84/06	quartz crystal			26.78	0.68	27.77			82.0	83.6	41.0	0.96	glue	1	skin+gel	41.0	point breakage in haft
Exp. 84/07	quartz crystal			28.28	2.3	31.03			82.0	84.2	40.0	2.49	glue	1	skin+gel	19.5	
Exp. 84/08	quartz crystal			26.29	1.65	28.16			83.5	85.3	39.9	3.22	glue	2	(1) skin+gel+bone; (2) skin+gel	(1) 45.5; (2) 21.8	point breakage in haft
Exp. 84/10	quartz crystal			26.79	3.36	30.58			83.5	85.2	38.5	4.81	glue	1	skin+gel+bone	0	de-hafted
Exp. 84/11	quartz crystal			28.27	1.01	29.54			83.5	85.5	42.1	0.76	glue	1	skin+wall	0	point breakage; dehafted
Exp. 84/14	quartz crystal			28.5	2.26	31			83.5	85.7	40.6	2.63	glue	1	skin+gel+bone+wall	0	point breakage; dehafted
Exp. 84/15	flint			33.46	2.91	33.12			83.5	86.5	41.5	2.02	glue	2	(1) skin+ gel; (2) wall	48.0	point breakage; dehafted
Exp. 84/24	flint			36.56		35.5			82.0	84.5	35.5	7.99	glue+sinew+glue	1	skin+gel+bone	10.5	point breakage in haft
Exp. 84/25	flint			34.1		34.1			82.0	86.5	37.0	7.23	glue+sinew+glue	3	(1–2) skin+gel; (3) skin+gel+bone	(1) 23.0; (2) 21.0; (3) 13.0 + point	point breakage; dehafted
Exp. 84/26	flint			33.59		33.59			82.0	84.0	37.0	5.95	glue+sinew+glue	2	(1) skin+gel; (2) skin+gel+bone	(1) 13.5; (2) 9.0	point breakage in haft
Exp. 84/27	quartz			30.49		30.49			82.0	86.5	40.0	3.76	glue+sinew+glue	1	skin+gel+bone	2	point breakage; dehafted
Exp. 84/28	quartz crystal			28.76		28.76			82.0	83.5	39.5	2.69	glue+sinew+glue	1	skin+wall	0	point breakage in haft
Exp. 84/29	quartz crystal			35.46		35.46			82.0	83,0	40.0	1.81	glue+sinew+glue	1	skin+gel	9.0 + point	dehafted
Exp. 84/30	quartz crystal			28.85		28.85			82.0	85.5	42.0	0.88	glue+sinew+glue	1	skin+gel+bone	10.3	point breakage in haft
Exp. 84/31	quartz			29.59		29.59			82.0	83.0	38.5	3.61	glue+sinew+glue	4	(1–3) skin+gel; (4) skin+gel+bone	(1) 14.0; (2) 16.0; (3) 18.0; (4) 17.0 + distal tip of point	point breakage in haft
Exp. 84/32	quartz			32.52		32.52			82.0	82.0	34.8	7.56	glue+sinew+glue	1	skin	0	dehafted
Exp. 84/33	quartz			30.45		30.45			82.0	86.0	40.3	3.14	glue+sinew+glue	1	skin+wall	0	point breakage in haft

Penetration depths in the target could only be measured for points that remained stuck in the target after the shot. When the point detached from the shaft inside the target, only the penetration of the shaft (and the remaining part of the point, if relevant) could be measured. The (fractured off) point length should be added to this value (indicated in the table as “+ point”).

**Fig 8:**
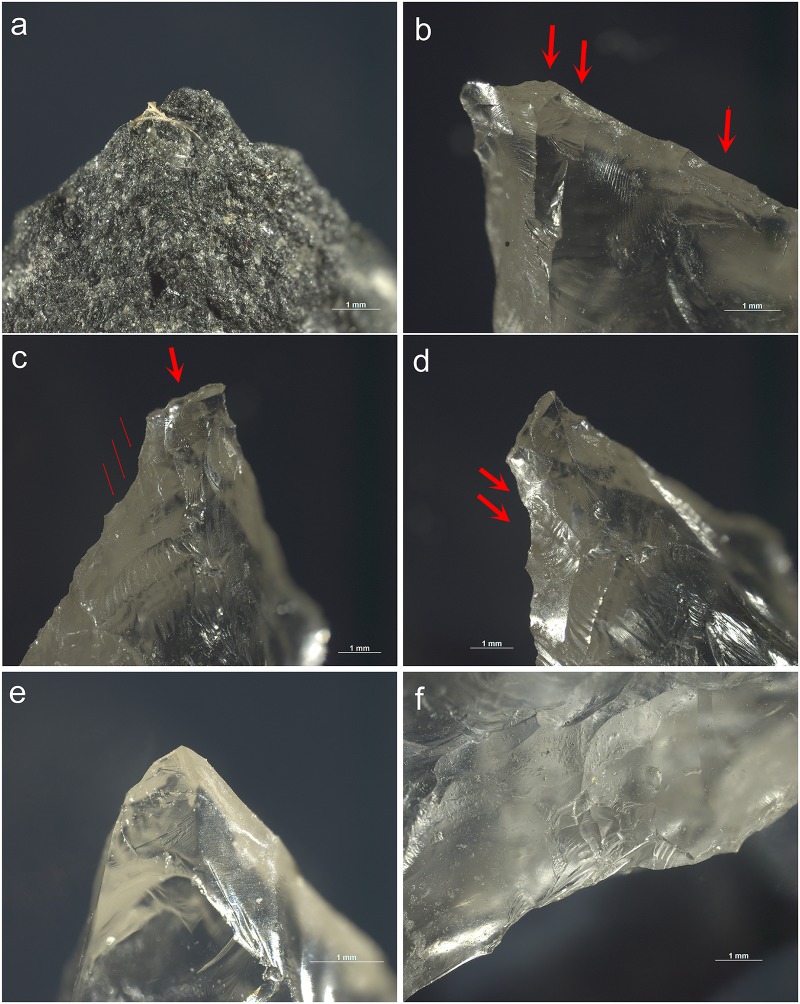
Examples of damage caused by impact as recorded on the experimental reference sample. **a)** step-terminating bending fracture on the apex of Exp. 82/135 (dolerite; x26.5); **b)** lateral step-terminating scarring on the ventral apex of Exp. 84/10 (x30.5); **c)** step-terminating spin-off initiated from the apex fracture on the dorsal distal tip of Exp. 84/10 (x23.5) (also lateral scarring is visible); **d)** elongated bending initiated and step-terminating scars on the lateral edge of the dorsal distal tip of Exp. 84/10 (x23.5); **e)** superposing step-terminating scars on the ventral distal tip of Exp. 84/7 (x40.5); **f)** superposing step-terminating scars on the right lateral edge of the dorsal distal tip of Exp. 84/7 (x26).

Fractures occurred as a result of the contact with the hide as well as the contact with bone. Given the robustness of the target, de-hafting occurred frequently (Fig 7). In general, the projectiles in quartz proved very resistant to damage when used as a projectile. The damage that was incurred consisted of small fractures or unifacial scars and crushing (Fig 8). The projectiles in dolerite showed clear signs of damage, including a lot of abrasion of the edges. The edge-wear susceptibility of the edges was of course reduced by the bifacial nature of the pieces. It has been observed experimentally (cf. [53]; TraceoLab experiments) that bifacial points are more resistant to damage than unretouched or unifacially retouched points. Also this experiment showed that bifacial points suffered from relatively little damage in spite of the experimental setting that was intended to intensify damage formation by increasing the chances of contact with bone.

## Results

Evidence of tool production, use and hafting was observed in the form of residues and wear traces. In spite of the age of the material, residues were very well-preserved, which concurs with previous studies at Sibudu [54–56] and corresponds to the general pattern of high quality preservation of organic material in South African cave and rock shelter sites. Interpretations are based on a combination of different residue types related to a single type of worked material (e.g., combination of collagen, animal tissue and tendons in the case of animal-related use), which significantly strengthens the link of a residue with a tool’s use [57]. Residue causality is further confirmed through the combination with an independent wear analysis (cf. [58]).

For the present study, the analytical research revolved around three main questions: 1) what is the evidence for the use of pressure flaking to serrate the bifacial pieces? 2) what were the serrated points used for? 3) how were the serrated points used, and in particular, is there evidence of hafting?

### Evidence for pressure flaking

To test the hypothesis that the serration was created by pressure, we examined different types of data corresponding to both the effect of the technique used and the direct contact between the compressor and the stone tools: 1) evidence on the bifacial pieces (the negative traces), 2) evidence on the shaping flakes (the positive traces), 3) wear and residue evidence from the manufacturing process (the remnant traces). We believe that only the combination of these different lines of evidence allows the demonstration that pressure technique was used or not. In addition, further support was obtained by examining the bone tool assemblage, by producing a set of experimental replications and by comparing with available published data on the subject of pressure flaking (see [59]).

However, we have to acknowledge the following biases with regard to our study in comparison to published data:

1. Most of the current literature about pressure flaking deals with raw materials such as flint and silcrete (e.g., [5]). Differences in rock properties (influencing the way they fracture) should not be overestimated in the sense that pressure can be applied to all sorts of raw material [60]. However, the way in which technical scars are recorded can vary significantly between raw materials and rock properties may blend the usual technical criteria used to establish the diagnosis. In sum, potential differences have to be expected with regard to the nature of the rocks that were shaped. In this regard, the experiments with quartz and dolerite helped us to re-assess and “calibrate” our list of criteria.
2. Secondly, most of the literature deals with pressure flaking and not formally with pressure notching. Pressure flaking aims at regularizing an edge and thinning the sections of the tool, while pressure notching aims at serrating an edge. Both procedures share very similar characteristics but there are also variations. In the case of pressure thinning, the compressor has to be placed on the very edge of the blank that would have been initially prepared by abrasion in order to strengthen the contact. The force applied has to be in an angle that is more or less parallel to the plane of the blank. As a consequence, the flake will often have a small bulb and will have an elongated and regular morphology with parallel edges. By contrast, in the case of pressure notching, the compressor has to be placed slightly inside the edge and the applied force has to be at an angle more or less perpendicular to the plane of the blank [35,61]. This angle will influence the depth of the notch and consecutively determine the morphologies of the serration flakes (thickness and breadth of the butt and overall morphology).

We also have to acknowledge that discrimination of the use of pressure may be difficult due to similarities with soft hammer percussion (cf. [5]). However, given the difference in the type of contact (percussion or pressure), both techniques display different stigmata from the preparation. The use of soft hammer percussion is associated with a tangential percussion which requires an abrasion that is in theory wider and more intense than for pressure, as the exact location of the contact point is more uncertain and the initiating force is more dramatic. This contrasts with a localized and more regular progression in initiating the force with pressure technique. Furthermore, the resulting waste flakes between percussion and pressure vary accordingly, showing differences in the butt morphology, the presence/absence of a lip and a contact point, the characteristic of the bulbs (not extensive but pronounced for pressure versus no bulb for soft tangential percussion) and hackles on the bulb (but see [62]).

#### The serrated pieces

**Fig 9:**
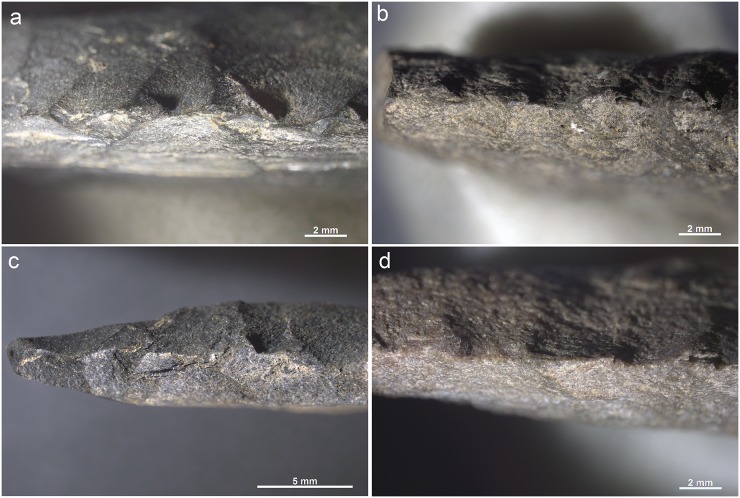
Details of the serration. **a)** point 535 (x12.4); **b)** point 546 (x12.4); **c)** point 1021 (x11.2); **d)** 1055 (x12.4)

Several lines of evidence support the use of pressure. Firstly, as based on previous studies of bifacial pieces, the size of the notches (width and depth) and their spacing is an important parameter [16,36,63]. The regularity of notches (within a single piece) indicates a clear control of the locations of the contact points as well as of the force that was applied (Fig 9). As noted previously, the assemblage of Sibudu has notches with a mean width of 3.2 ± 1.7 mm and a mean depth of 0.5 ± 0.3 mm. These measurements indicate that the tool used to initiate the removal of the notches should have been of a relatively small size. The key relevant information, however, is the variability in the dimension of the notches. Interestingly, the standard deviation is lower when we consider measurements for individual pieces and sample sets. Indeed, 21 on a total of 25 pieces have notch width measurements with a standard deviation less than 1.7 mm, including 11 pieces with a standard deviation less than 1.1 mm. Similarly, 17 from a total of 25 pieces have notch depth measurements with a standard deviation below 0.3 mm. Both observations suggest that the serration was more regular per piece than between pieces, which indicates a strong internal homogeneity and control of the notches per piece.

A second argument for the use of the pressure technique comes from the bifacial serration itself. Indeed, creating a notch by percussion requires the edge of the blank to be convex (or rectilinear) in order to ensure a good contact with the hammer. One advantage of the pressure technique is to be able to detach removals in areas that cannot be reached by percussion, such as concave edges. On a few bifacially serrated pieces (Fig 9), we see that the serration is symmetrical on both faces, meaning that the fracture of the last notch was initiated within the concavity of the previous one.

Additional evidence relates to the negatives of the serrating removals themselves. These show small hackles and negative bulbs, which are indicative of a narrow contact point. Furthermore, they exhibit parallel edges and a feathered termination [5,7].

Further support comes from the presence of minor abrasions on the edges of the points, on both finished and unfinished forms. Such minor abrasion is applied before pressure flaking to create an artificial placement of the compressor tip behind the edge. Similar abrasion occurred on some experimental points.

**Fig 10:**
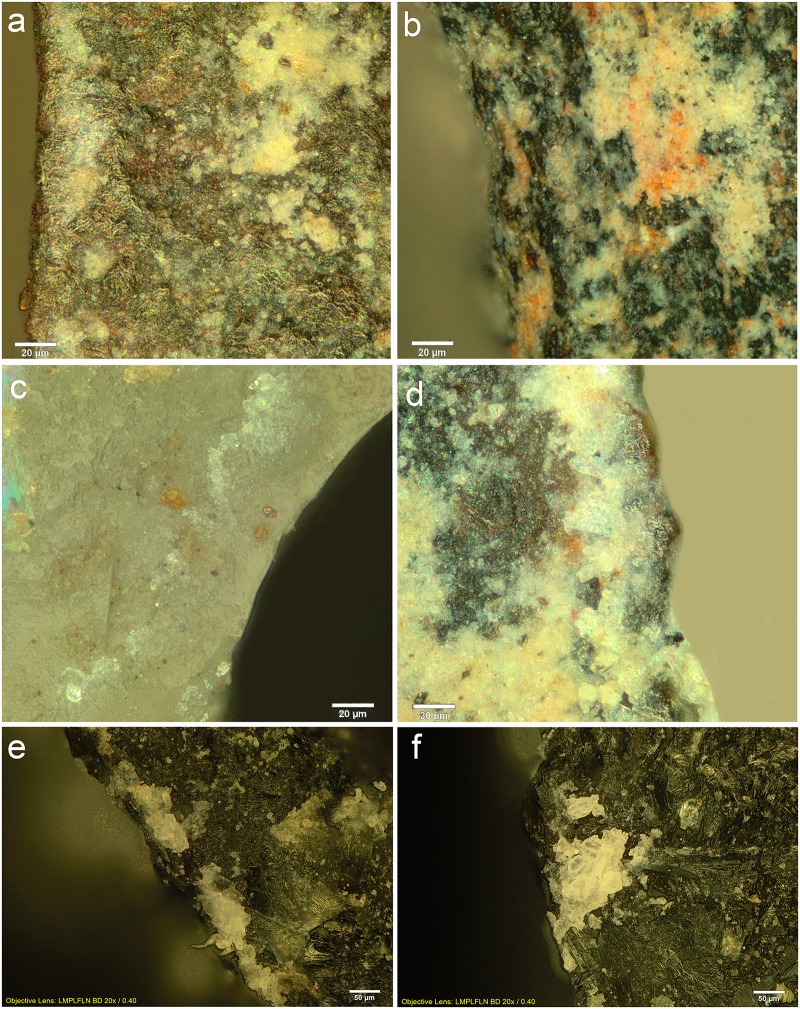
Examples of residues related to pressure flaking with bone. Retouch residues on: **a)** the dorsal left distal edge of 551 (x500, dark field); **b)** the dorsal left distal edge of 1021 (x500, bright field); **c)** the dorsal right medial-proximal edge of 1803 (x500, bright field); **d)** the ventral left medial edge of 875 (x500, dark field); **e)** and **f)** examples of bone retouch using pressure flaking from comparative reference material (f after washing) (x200, dark field).

Finally, bone residues occurred in association with the negatives of the serration on 11 of the 15 examined points, in particular on their distal and medial sections (Table 10). Good examples were observed independent of raw material (e.g., distal edges of 551-hornfels and 1021-dolerite, medial edges of 1803-quartz and 875-hornfels) (Fig 10).

**Table 10:** Summary of residues on the serrated pieces.

Tool number	Section	Face	Animal									Plant					Mineral				*Sedimentary particles (indet*.*)*	*Micro-charcoal/ TA palynofacies*	*Tissue (indet*.*)*	Stain
			*Tissue*	*Fibre*	*Sinew/Connective tissue*	*Skin (ctm*.*)*	*Hair*	*Proteinaceous residue*	*Blood*	*Fat/Lipid*	*Bone*	*Tissue*	*Fibre*	*Resin*	*Starch*	*Phytolith*	*Vivianite*	*Hematite*	*Crystal*	*Mg/Mn*				
**1170**	Distal	D	**R**T**U**	U	RU	R?U		**R**	R	**R**	RU						U	R?U			RU	U	RU	
		V	**U**	U	U	R?U		R		R	U				U	U		RU			RU	U	U	
	Medial	D	RT	**R**	R?				R	RT	T	T	T	U?	**U**	T		R			RU	U	RTU	
		V			R									U?							U	U	RTU	
	Proximal	D	RTU	**R**	R			R		R	R	RTU	RT	R?T?U	TU	TU	T	R			RU		RTU	
		V	U	U	R							**RTU**	R	RT?U	TU	U		R?			RU	U	RTU	
**1075.1**	Distal	D	RTU	T	U?	RU				U	R?T?	T	T	RT?	T**U**	T		R?		R	RTU	U	R	
		V	U		U?	U							T	R?	**U**			R?			RTU	U		
	Medial	D	TU	T	TU?	U	T	R			R?T	TU	R**T**U	R?T?U?	RT**U**		U?	R?T?		R	RU	RTU	U	
		V	U		U?			R?				U	U	R?U?	U		U?	R?		R	RU	RTU	U	
	Proximal	-																						
**535**	Distal	D	RT		R	R?		R	R	R	R							R?					RT	
		V	R			R		R	R		R?												RT	
	Medial	D	R					R	R?		RT			? (lin)									RT	R
		V	RT						R?	**R**T	RT	R**T**	**RT**	**R**	**T**		T	RT			**RT**	R		
	Proximal	D										R		**R**										
		V	RT									R *	**RT**	**R** *							**RT**	R		
**1803**	Distal	-																						
	Medial	D	RU			RU				R	R	U		R?	RU	U		RU		R?	RU	RU	RTU	
		V	RTU		T?	U		R?	R?	T	R	TU	T	R	U	TU		RU		U	RTU	RU	RTU	
	Proximal	D	U			RU					R	U		R?	U	U	T?	U		R	RU	RU	RTU	
		V	U			U		R?			R	U		R?	U	U		U	TU (CaCO_3)_	R	RTU	U	RTU	
**1804**	Distal	D	**RT** *	R	R	R		**R**	R? *	R	RT				R**T**		RT				R *		RT	
		V	RT			R		**R**		R											R		RT	
	Medial	D	**RT**	T	R	R		R	R?	R		**RT**	T	R*	**R**T			R *	NaCl*		R	**R**	RT	
		V	**RT**			R		R				**RT**			T								RT	
	Proximal	-																						
**551**	Distal	D	R					**R**		**R**	R										R		R	
		V	**RT**					**R**		**T**	T			R?			T				RT	RT	R	
	Medial	D	R *					R		R	R?			**R** *				R			R		RT	
		V	R		R?						R?	R	**T**	RT		T	T	R			**RT**	RT	RT	R
	Proximal	D												R				R			R			
		V								R		R		R	R			R?			**RT**		R	R
**665**	Distal	D			**R**			R	R	RT	R?	R?									RT		RT	
		V	**RT** *	**RT**	R *		T	**R** *	R *	RT											RT		RT	
	Medial	D			R			**R**	R	T	R?T	T?			T			**R?**				R	RT	
		V	**RT**		R			**R**	**R**	**RT**	R?							T?	NaCl? *				RT	
	Proximal	-																						
**546**	Distal	-																						
	Medial	D	U		U	**RU**				**T**	U	U	U	U?	R?U	U		R?		R	RTU	U	RU	
		V			U	**RTU**				**T**	U	U	TU	U?	U	U		R?			RTU	U	RU	
	Proximal	-																						
**877**	Distal	-																						
	Medial	D	**RT**			RU		R	R?	U	R?U?	RU	TU	R?U?	RU	U	T	RU		R	**RTU**	U	RTU	
		V	**RTU**		RT	RU				U	U?	RU	TU	U?	U	U		RU			**RTU**	U	RU	
	Proximal	D	RT**U**	U?	U			R		U	TU	R**U**	RTU	**R?U**	R?U	U		RU	**TU** (CaC_2_O_4_)		**RTU**	U	RTU	
		V	R**U**	U?	U	RU	T			U	**TU**	**RU**	U	RTU	RU	U	T	RU	**TU** (CaC_2_O_4_)		**RTU**	U	RU	
**1889**	Distal	D	RT**U**			RU		R	R?	R?	R?U?			R	RT		RT	R?U?		R	RU	R		
		V	U			U					U?									R	RU			
	Medial	D	U			U			R?		U?			R?				R		R	RU			
		V	U			U					U?									R	U			
	Proximal	-																						
**1136**	Distal	D	**RTU**			U		R	R?	U?	R?	U	U	U?	R?TU	TU	U	R?			**RTU**	T**U**	RTU	
		V	RU			RU		R	R?	R	R	U	U		U	U	U	R?			**RU**	**U**	U	
	Medial	D	**U**			R?U		R	R?		R	U	U	R?U?		U		R?			**RU**	**U**	RU	
		V	RU			U		R	R?	R	R?	U	U	RU?		U		R			**RU**	R**U**	U	
	Proximal	D	U	U?							R?	U	**U**	R?U?	U	U		R?			U	**U**	RU	
		V	U	U?						R?	R?	U	**U**	R?U?	U	U		R			U	**U**	RU	
**875**	Distal	D	RTU		RU	RU	RT	R	R? T?	RT		U			T	U	T	RU?			**RTU**	U	U	
		V	U		U?	RU		R		R	RTU	U				TU		U?			**RTU**	U	RTU	
	Medial	D	TU		T?U	RU		R			R	RT		T?				RU?	**TU** (CaC_2_O_4_)	Mn *	**RTU**	U	U	
		V	U		U?	RU	T			T	R	T	T					U?			U	U	RU	
	Proximal	D	U		U						RU?	U	U	U		U		U?			U	U	**R**TU	
		V	**T**U		U					R?	U?	U	U	R?U		U		R?U			RTU	U	RU	
**632**	Distal	-																						
	Medial	D	RU		U?	U		R		R	R?	U	U	**RT?**	R?U	U	T	**R**U?			RTU	U	**RTU**	
		V	RU		RU?	U		R	R?	R	R?	U	U	R	U	U		RU?			RTU	U	U	
	Proximal	D	R			R	U	R		R	R?	TU	TU	**RT?U?**	U	U	TU	RU?		R	RTU	U	U	
		V					U				R?	U	U	**R?U?**	U	U	U	RU?			RTU	U	**RTU**	
**845**	Distal	D	**RT**U	RU	T			R	R?	R	RTU		R?		U				T**U** (CaCO3, CaC2O4)		**R**	**TU**	T	
		V	**RT**U	RU	**RT**			R	R?	R	RTU				U				T**U** (CaCO3, CaC2O4)		**R**	**TU**	T	
	Medial	D	**RT**U	TU			U	R		R	RTU		T	U?				R	**T** (CaCO3, CaC2O4)		**RT**	**TU**	TU	
		V	**RT**U	TU	T		U	R		RT	U		T	T?U?			T	T	T (CaC)3, CaC2O4)		**RT**	T**U**	TU	
	Proximal	-																						
**1021**	Distal	D	R**T**U	R**T**	R			R		**R**	RT				U	U	T		T		**RTU**	**TU**	RTU	
		V	R**T**U	R**T**				R		**R**	RT				U	U	T		T		**RTU**	**TU**	RTU	
	Medial	D	RTU	**T**U			R?T	R		R	R		T	R?U	U	TU		R	T		**RTU**	R**TU**	RTU	R
		V	RTU	**T**U				R			R		T	R?U	U	TU		R	R (olivine) T (CaCO3)		**RTU**	**TU**	RTU	R
	Proximal	D	R							R								R			**R**		R	R
		V	R		R						R							R			**R**		R	R

R (reflected light analysis of tool surface), T (transmitted light analysis of pipette samples), U (transmitted light analysis of ultra-sonic bath residues,

* (presence of residue confirmed with SEM–EDS),

**bold type** (indicates residues are very common to abundant).

#### The shaping and serration flakes

In addition to the 25 bifacial serrated pieces, we also integrated the analysis of the flakes <30 mm from the sieved buckets within this study in order to sort the retouch/shaping flakes, as well as the purported serration flakes. We decided to focus only on the flakes made from quartz as it is one of the primary raw materials of the population of serrated pieces (n = 7). Also, the visibility of technical damage is more clearly visible on quartz compared to, for instance, dolerite and quartzite. In total, 76,802 small flakes have been screened from layers Adam to Chantal.

Square C4 and layer Bart (GSS) have been chosen here to illustrate the frequencies of retouch, shaping and eventually serration flakes that we recorded within the flakes <30 mm category (work in progress). 279 small flakes made from quartz of a total of 2966 originate from square C4 and 26 (9.4%) of these have been identified as shaping flakes, including 3 serrating flakes. Because of the general overlapping nature of the criteria used to distinguish shaping flakes from informal small flakes, and because of the brittle nature of small quartz products, the proportion of shaping flakes in general, as well as the proportion of serrating flakes in particular, should be taken as a strict minimum. While it is not always possible to distinguish between retouch flakes and shaping flakes, this aspect is of less importance here, as the quartz tools are predominantly composed of bifacial pieces within the studied assemblages (77.6% of all quartz formal tools are bifacially shaped).

Shaping flakes *sensu lato* have been defined based on their morphology, profile, platform type as well as form and number of dorsal removals (see for criteria Soriano et al., 2009). Shaping flakes and purported serrating flakes were distinguished based on the thickness of their butt and their overall morphology. We acknowledge that such distinction should refer more closely to the experimental collection. However, for the present paper, the aim was (1) to identify shaping flakes whose morphology suggests their removals created a notch on the edge of the tool, and (2) to describe the overall technical characteristics of these ‘notching’ flakes. They were all examined under an Olympus binocular stereomicroscope with a magnification up to x56; pictures were taken with a Canon EOS and were processed with the software Helicon Focus. In total, 24 purported serration flakes coming from layers Adam to Bea have so far been identified.

**Fig 11:**
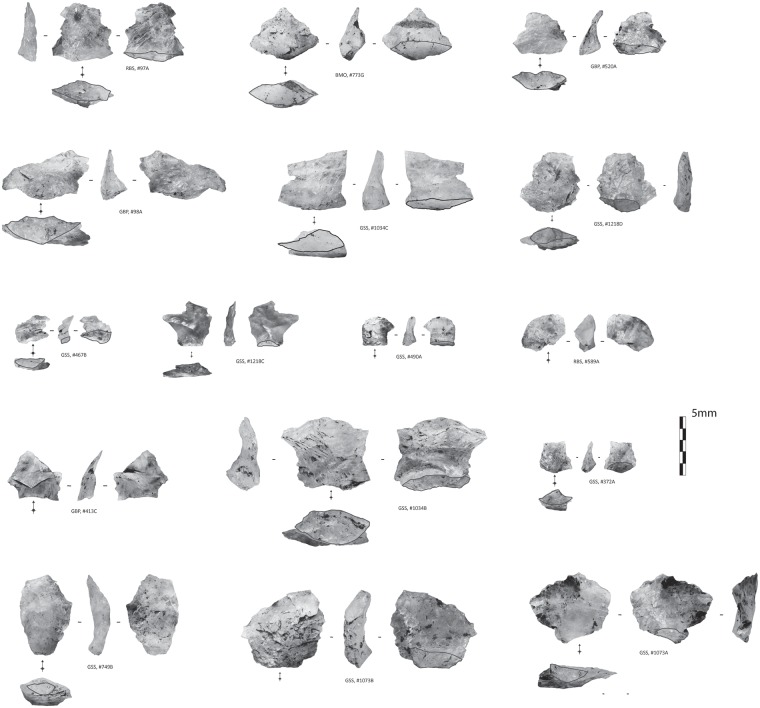
Sibudu basal deposits—Serration flakes.

The so-called serrating flakes (Fig 11) exhibit butts with a mean width of 3.9 ± 1.9 mm (n = 21) and a mean thickness of 1.5 ± 0.6 mm (n = 24). These data are in accordance with the measurements of the notches taken directly on the bifacial serrated pieces (cf. infra). These purported serrating flakes have butts with predominantly lenticular morphology (n = 16) but the shape of the butts’ surfaces, which are either straight (n = 12), convex (n = 4) or concave (n = 2) is more variable. For the convex and concave examples, the presence of the contact point within a concavity suggests the shaping flakes record a phase of symmetric bifacial serration. By contrast, one example has a contact point directly on the convexity of a ridge, suggesting a phase of asymmetric bifacial serration.

The morphology of these purported serrating flakes is short, with a length/width ratio of 0.7 (n = 19), dominantly made of parallel edges (n = 8) or alternatively convergent (n = 5) or divergent edges (n = 1). A minority of the sample has clear bulbs (n = 9), characterised by being short but relatively pronounced. Neither contact points, as inferred from the presence of incipient cones on the butts, nor the presence of a lip, have ever been observed. Interestingly, abrasion of the overhang of the butt is rare (n = 2) suggesting that abrasion was not a prerequisite condition for notching. Shattered bulbs (initiating from the butt) (n = 2) and bulbar shatters (not initiating from the butt) (n = 4) are both very rare but we often observed the presence of parasite shatters (initiating obliquely to the axis of the flake) (n = 8), that might either relate to the nature of the raw material or to the presence of an opposite force while the flake was detached (for example, the force of a pressure tool that will be slightly opposed to the force applied by the hand holding the piece).

The combined evidence based on the purported serrating flakes is in accordance with the use of a pressure tool to create notches on the bifacial blanks. By contrast, the population of small and formal shaping flakes often present impact points on the butt as well as small incipient cones that suggest the use of soft (stone) hammer percussion. However, we acknowledge that some of the formal shaping flakes have features that could be from pressure as well. Either some formal shaping flakes are associated with notching (but were not classified as such in the present study) or pressure was also used to shape the quartz bifacial pieces at a previous stage. Further work is required to better classify and evaluate the role of pressure within the present assemblage.

#### Bone tools used as compressors

**Fig 12:**
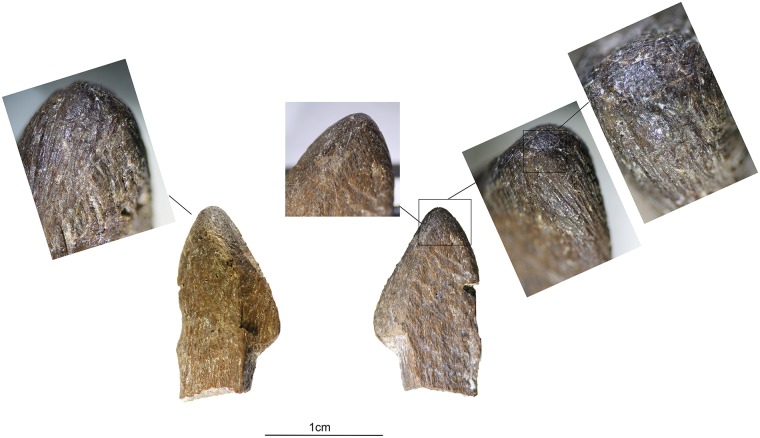
Bone tool A5-690-3 from layer Casper. Wear traces on tip from a use as compressor in pressure flaking.

**Fig 13:**
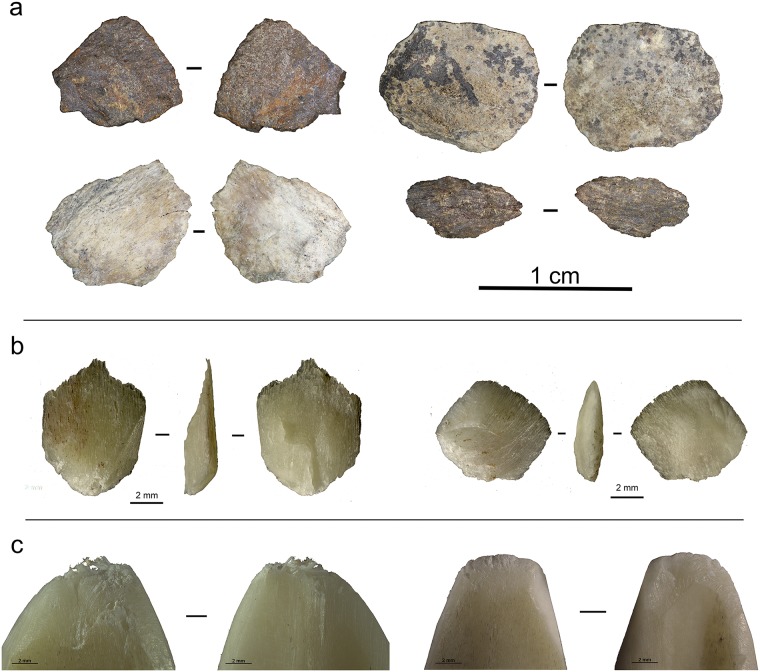
Bone flakes. **a)** archaeological examples of flakes that possibly detached from a bone compressor during its use in pressure flaking; **b)** experimental bone flakes detached from a bone compressor during its use in pressure flaking; **c)** damage on the tip of experimental bone compressors from use in pressure flaking.

In accordance with our aim to combine multiple types of data to identify the use of pressure technique, we also examined the bone tools of the corresponding archaeological assemblages. One bone fragment was identified in layer Casper that is interpreted as a bone compressor based on the macro- and microscopic evidence of use (Fig 12). The wear characteristics consisting of crushing, small fracture negatives and striations are entirely comparable to the experimentally-used bone compressors (Fig 13). In addition, a number of small bone flakes match the morphology and characteristics of the experimental bone flakes that occasionally detach from a bone compressor during use (Fig 13). Such characteristics are a rectangular to trapezoidal shape, a crushed platform, a small bulb, a straight profile and unidirectional dorsal removals.

**Fig 14:**
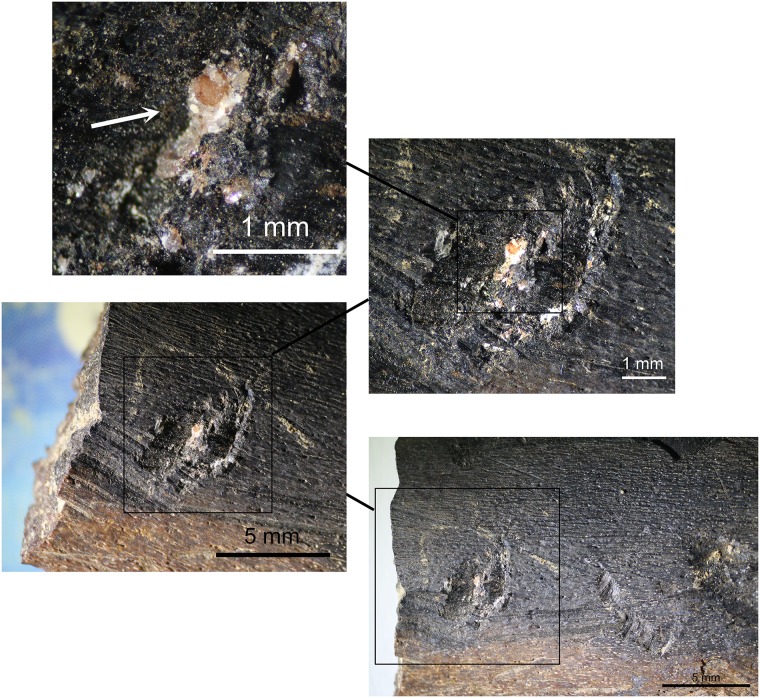
Bone percussion tool A5-598 from layer Casper with encrusted quartzite flake fragment.

Notably, a bone percussion tool was also identified from the same layer as the bone compressor fragment. Remarkably, this had a quartzite flake fragment still embedded in the bone (Fig 14). This tool is probably mainly associated with the quartzite bifacial tools discovered in the same layers, but also one serrated bifacial point in quartzite was identified for layer Bea just above (Table 6). This further emphasises the important role of bone in the stone tool production cycle of these assemblages.

Building on this direct evidence, we may also try to establish a closer comparison between the purported bone tool compressor and the notches observed on the serrated pieces. Indeed, the variability in the width of the notches clearly concurs with the width of the extremity of the bone tool compressor. From this and the dimensions of the notches, it is expected that the compressors would have a contact point varying between 2 and 4 mm. This does indeed concur with the bone compressor found in the archaeological collection (measured at 1mm distance from outer tip, the contact point is 2.5 mm wide).

#### Pressure flaking to serrate points

If we combine all the data discussed above, the most parsimonious explanation is that the toolmakers of Sibudu used pressure flaking for the production of the notches on the serrated points. On the one hand, the regularity of the notches, the bifacial symmetry of the serration and features of the serration removals on the pieces themselves, as well as the diagnostic characteristics of the by-products indicate the use of pressure technique. On the other hand, the presence of compressors in the bone assemblage and the residual evidence reinforce the interpretation that the serrated pieces could have only resulted from pressure flaking. This is in accordance with previous studies on serrated pieces (e.g., [35,36]) that recognize the use of pressure flaking as the only technique possible to create small and regular indentation.

### Evidence of projectile use

On 14 points or point fragments, impact-related damage was observed in association with animal residues and wear features that confirm the points were used as elements in hunting weapons. The distribution of the evidence confirms them having been mounted as tips on shafts. Use-related residues occur in the distal sections of the stone tools and mainly consist of proteinaceous and related residues including tissue fragments, blood, fat and bone, which indicate usage on animal material. The encrustation of several residues within impact-related scarring confirms the tools were used as hunting weapons.

Seven complete projectile points were identified, four of which were identified with a high level of confidence (one of these has a small proximal fracture) and three with moderate confidence (Table 11). In addition, three projectile fragments were identified with a high level of confidence: a base fragment and two tip fragments. The high degree of confidence of these interpretations is based on a combination of different wear features and residues (see below) that are all in agreement with respect to their cause. Another tip, a base fragment and two medial fragments were identified as part of projectiles with poor to moderate confidence due to fewer lines of evidence.

**Table 11:** Conclusions of the functional analysis.

Layer	Unit	ID	Complete / Fracture	Residues		Wear traces		Final interpretation	CL
				Abundance	Interpretation	Interpretation	C.L.		
GSS	B4	1170	complete	medium	used	projectile tip	4	projectile	4
GSS	C4	1075.1	distal fragment	low	uncertain	projectile tip	3	projectile tip	3
GBP	A4	535	complete	medium	used & hafted	possibly used projectile	1–2	projectile	2
GBP	B4	1803	basal fragment	medium	uncertain	projectile base	2	projectile base	2
GBP	B4	1804	distal fragment	high	used & hafted	projectile tip	4	projectile tip	4
GBP	B5	551	complete	medium	used & hafted	projectile tip	4	projectile	4
GBP	B5	665	small proximal fracture	high	used	projectile tip	4	projectile	4
GBP	B5	546	medial fragment	low	uncertain	double fracture in projectile use	2	projectile fragment	2
GBP	C5	877	basal fragment	medium	uncertain	projectile base	4	projectile base	4
GBP	C5	1055	medial fragment	0	0	possibly double fracture in projectile use	1	projectile fragment	1
LBC	B4	1889	distal fragment	low	uncertain	unused	2	unused	2
LBC	B5	1136	complete	medium	used	projectile tip	4	projectile	4
LBC	B5	875	complete	medium	used	possibly used projectile	1	projectile	2
LBC	B5	748	complete	0	0	projectile pre-form, not well-preserved	2	unused	2
LBC	A5	632	basal fragment	medium	uncertain	fracture in production (shaping)	2	unused	2
PSS	A5	845	distal (medial) fragment	medium	used	used	2	possible projectile	2
COP	A5	1021	complete	low	used	used & hafted	2	possible projectile	1

Use-wear and residue results combined. (C.L. = confidence level of the interpretation on a scale of 0 –uncertain to 4 –certain)

The remaining points comprise one distal fragment, one basal fragment and one complete point. These pieces seem to have remained unused: they were mostly preforms fractured during production. It cannot be entirely excluded that some of these fragments fractured in use, but there is insufficient evidence from use-wear traces to support this with any degree of confidence.

**Fig 15:**
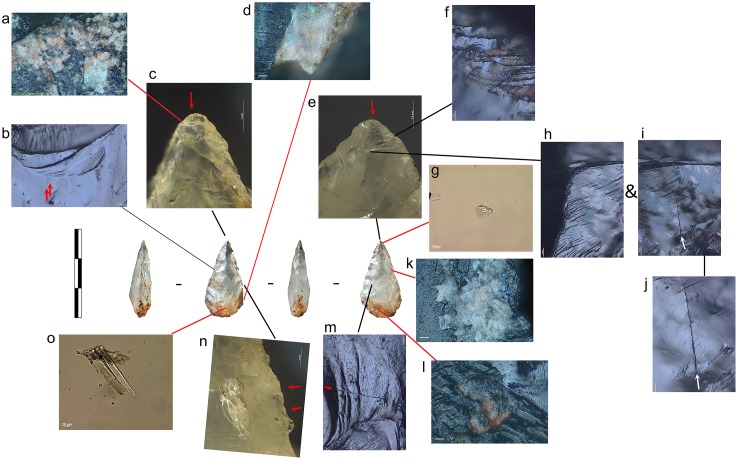
Functional evidence on point 1170 showing examples of impact wear, MLIT and residues. **a)** proteinaceous residue with animal tissue fragments and possibly blood (x500, bright field); **b)** MLIT’s associated with the termination of a scar caused by impact on the left lateral edge (x500, bright field); **c)** step-terminating bending initiated scar on the apex attributed to impact (x52); **d)** large sheet of sinew-like tissue on edge possibly use-related or from retouch or binding (x500, dark field); **e)** step-terminating bending initiated scar on apex attributed to impact (x92); **f)** residue compacted in the termination of the scar depicted in (e) (x500, bright field); **g)** small bone fragment (x400, transmitted light); **h)** abrasion on the termination of the scar depicted in (e) and attributed to friction upon impact (x200, bright field); **i)** linear surface feature close to but not associated with the termination of the scar depicted in (e) and due to the structural properties of the raw material (not use-related) (x200); **j)** detail of the linear surface feature (due to structural properties of quartz crystal and not use-related) (x500); **k)** animal tissue fragments probably from retouch but may also be from binding or use (x500, bright field); **l)** resinous-like adhesive residue (x500, dark field); **m)** friction striation on medial surface, possibly due to hafting (x100); **n)** cracked superposing step terminations of bending initiated scars (x35); **o)** articulated fibres associated with stellate trichome cf. *Pavonia* sp. (x400, transmitted light); (scale bar on residue images 20 μm).

**Fig 16:**
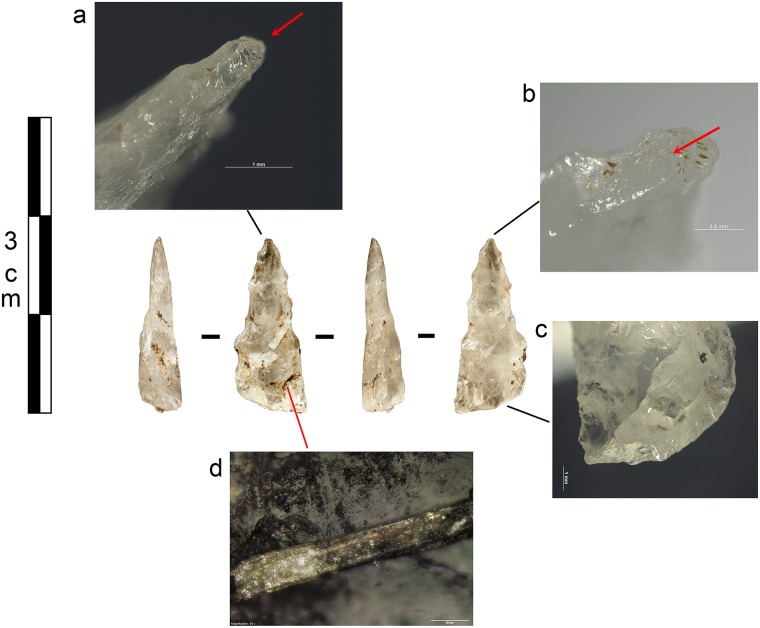
Examples of wear traces and residues on tip fragment 1075–1. **a)** small burination associated with small bending initiated hinge-terminating tip fracture (x58); **b)** small step-terminating spin-off associated with tip fracture (x77); **c)** medial fracture, complex fracture plane with multiple combined initiations (x21); **d)** compressed fibre with birefringent lipid inclusions (x500, bright field, polarisation)

**Fig 17:**
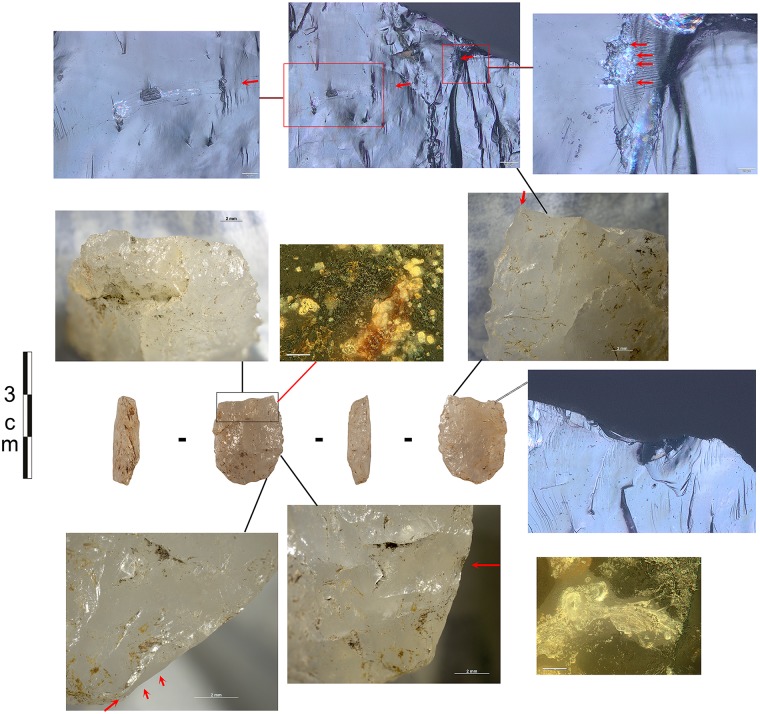
Examples of wear traces, MLIT’s and residues on basal fragment 877. **a-c)** MLIT’s associated with damaged fracture edge (a: x200; b: x100; c: x500, bright field); **d)** transversal bending initiated fracture terminating in a steep step (x11,2); **e)** fatty red-orange residue, possibly adhesive (x1000 dark field); **f)** secondary removal associated with transversal fracture (x11,6); **g)** obliquely initiated edge damage on fracture edge (x200, bright field); **h)** small elongated removal (cf. “burination”) with associated spin-off (x22,5); **i)** step-terminating bending initiated scar cross-cutting shaping retouch (x17,6); **j)** connective tissue wrapped around the edge (x1000, bright field).

The projectile evidence consists of a combination of different impact-related fractures and removals on the tips and lateral edges of the points (Table 12; Figs 15–17). This is in spite of the fact that bifacial points are more resistant to scarring as also demonstrated experimentally ([53]; TraceoLab experiments).

**Table 12:** Summary of the main wear features as observed on the used serrated points.

Tool ID	Low magnification																		High Magnification	
	Tip											Burination	Lateral		Proximal / Medial					
	Fractures				Unifacial scarring				Partial tip "explosion"	Associated spin-off	Tip crushing		Step/Hinge terminating scars	Crushing	Edge Damage (counter-pressure)	Burination	Fracture	Associated damage (to fracture)	MLIT	Other
	bending initiation				bending initiation															
	snap	feather	hinge	step	snap	feather	hinge	step												
1170								multiple			present		multiple	present				0	yes	abrasion
1075–1			tiny							several, small, step-terminating		lateral initiated at tip, small	multiple	present	present (lateral)		medial, transversal, complex plane (various initiations)	damage, various spin-offs	(GC)	(GC)
535								medium size, on limit with fracture	intense lateral damage, all step- and hinge terminating				intense lateral damage as part of explosion		present on base				yes	striated polish
1803	-	-	-	-	-	-	-	-	-	-	-	-		present	step-terminating scarring		medial, transversal fracture, also small fracture on base	yes, on lateral edge (medial) and on base	-	friction striations from fracture under impact in haft seem present (preservation not ideal)
1804						multiple, small		multiple, small			present		multiple	present		several, lateral, associated with fracture	medial, transversal	scarring, spin-off	(GC)	(GC)
551								multiple, small				lateral, small, step	multiple		present (lateral)	butt, lateral initiation, step-terminating			(GC)	(GC)
665								multiple, small	intense lateral damage				multiple	present			proximal, transversal	crushing, scarring	(GC)	(GC)
546	-	-	-	-	-	-	-	-	-	-	-	-					double	large, step-terminating, associated with fracture	yes	friction BS/S associated, P within concavity
877	-	-	-	-	-	-	-	-	-	-	-	-	smaller scars, more related to hafting than impact		present (lateral)	lateral, initiated from damage	medial, transversal	spin-off, damage	-	friction striations from fracture under impact in haft
1055	large				-	-	-	-	-	step-terminating	-		associated with fractures		yes	yes, on fracture edge, perpendicular on edge	yes	yes, on both corners	possibly, difficult due to raw material	friction S seem present in association with fracture but difficult observation given raw material
1136	small									large, step-terminating			multiple	some	present (lateral)				yes	striations
875								multiple, large, bifacial	intense lateral damage, initated from tip; cf burinations				multiple		present (lateral)				yes (likely)	striations
845	large									yes, not well visible							snap, oblique initiation	yes		
1021		medium size											yes, large, explicit step, oblique orientation						yes	linear striations and polish

(damage patterns, polish—P, striations—S, bright spots–BS, abrasion, microscopic linear impact traces—MLIT’s). No high magnification analysis could be performed on pieces that were preserved for gas chromatography analyses as these could not be cleaned (marked as (GC) in table).

A pronounced difference was observed between the damage patterns on the points made from quartz in comparison to those made from dolerite or hornfels. Damage on the former was significantly more limited and primarily consisted of small unifacial scars and crushing, while significant damage was observed on the dolerite and hornfels points, including partial explosions of the tip. This corresponds to what was also observed experimentally and it is linked to the difference in hardness between the raw materials.

The occurrence of transversal fractures is another relevant element for points that may have served as projectiles. In the case of the Sibudu serrated points, this element needs to be integrated with the raw materials used, as these differ in their susceptibility to damage and fracturing. Only 3 of the points made from dolerite (n = 7) had transversal fractures, one of which was only partial (proximal), while only one of the quartz points (n = 7) remained complete. Reasons for such discrepancy is probably due to the overall point size differences in the two groups, as well as differences in raw material hardness and the crystalline structure affecting resistance to impact shock.

Also, it is documented experimentally that hafting has an influence on the occurrence of transversal fractures, whereupon the least resistant element fractures upon impact [47]. Stone tools that are securely fixed in their hafts, for instance with a strong glue, have a higher chance of fracturing in comparison to less securely fixed tools for which the hafting will either absorb the exerted pressure or fracture itself, in particular in the case of impact-rich activities. If the glue used is brittle (e.g., pure resin), fracturing upon impact is more likely in comparison to the use of more flexible glue (e.g., with added beeswax). Transversal fractures occurring around the haft boundary have to be distinguished from fractures in the distal section for which we experimentally observed that the hafting mode does not affect the type of fractures that are formed upon impact but only their frequency [48]. Given the size and raw material differences between the points, it is unlikely that the strength of the hafting arrangement is the primary determining factor to explain the transversal fractures: equal pressure on identically hafted points likely leads to transversal fractures on small robust points and intense damage and partial explosions on larger more fragile points. Based on the use evidence, it may thus be hypothesised that the observed differences are a likely consequence of size or raw material variation, even though one cannot exclude either a more robust hafting mode for the points in dolerite and hornfels or a different projecting mode involving more pressure upon impact (but an equally strong hafting mode). The hafting evidence will shed further light on this issue (see below).

**Fig 18:**
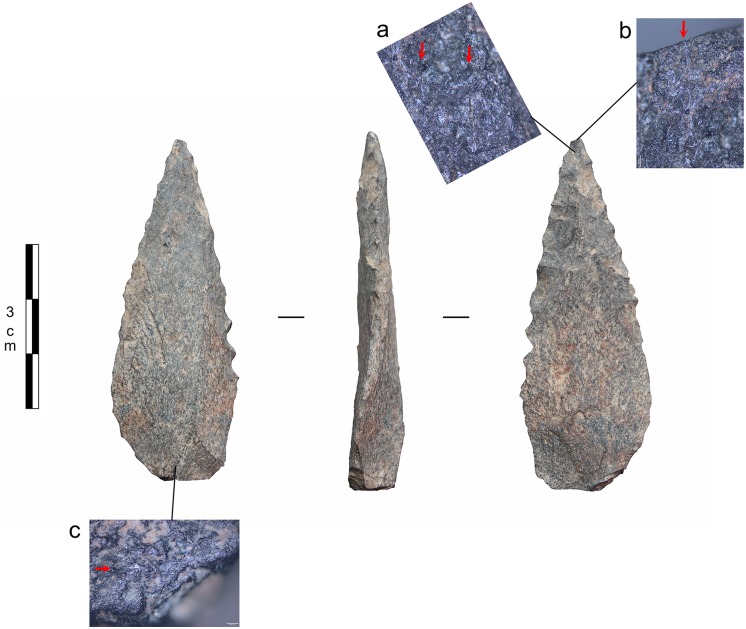
Examples of wear traces on point 1021. **a)** MLIT on ventral distal point (x100, DIC); **b)** striations (MLIT’s) associated with edge damage, both attributed to counter-pressure within haft upon impact (x100, DIC); **c)** MLIT’s on ventral distal point (x100, DIC).

Under high magnification, linear features as well as polishes and rounding are visible. In particular the linear features, including MLIT’s (microscopic linear impact traces; [14,64]) are relevant as they are formed by the friction of the fractured tip/flake against the point surface during insertion into the animal (Figs 15–18). They are direct evidence of the causal link between the fracture and the exerted longitudinal pressure upon impact. MLIT’s were observed on several of the analysed points.

**Fig 19:**
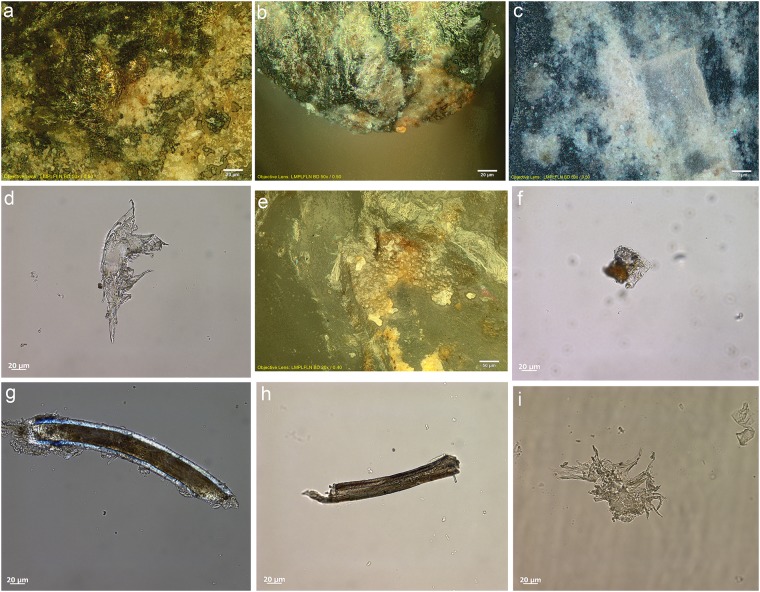
Examples of use-related animal residues. **a)** blood-like residue and bone on the dorsal medial surface of 1136 (x500, dark field); **b)** animal tissue embedded in proteinaceous residue on the ventral distal tip of 551 (x500, dark field); **c)** sinew on the dorsal right distal edge of 1804 (x500, dark field); **d)** torn animal tissue from the distal surface of 1804 (x400, transmitted light); **e)** adipose skin tissue on the dorsal left medial surface of 1804 (x200, dark field); **f)** fat globules in tissue from the dorsal medial edge of 875 (x400, transmitted light); **g)** hair fragment from the dorsal distal surface of 875 (x400, transmitted polarised light); **h)** hair fragment from the dorsal distal surface of 1075.1 (x400, transmitted light); **i)** torn animal tissue from the dorsal distal to medial margin of 1889 (x400, transmitted light).

**Fig 20:**
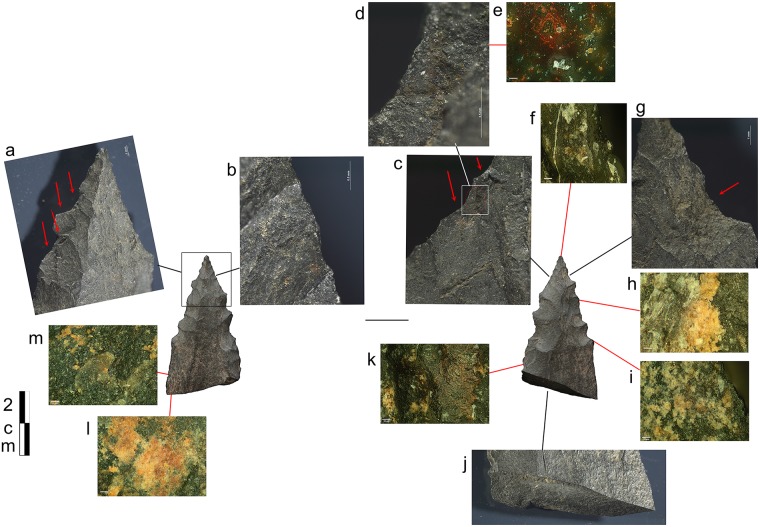
Wear traces and residues on point 665. **a)** multiple step-terminating scars on lateral edge of tip (x13.6); **b)** bending initiated scar with red residue (x84); **c)** bending initiated step-terminating scar with blood residue (x37); **d)** detail of (e) (150x); **e)** blood residues and tissue fragments (x500, bright field, polarisation); **f)** tissue, fibre and proteinaceous residue (x500, dark field); **g)** black residue within bending initiated step-terminating scar (x27.5); **h)** proteinaceous residue and smeared connective tissue (x500, dark field); **i)** abundant proteinaceous residue and tissue (x500, dark field); **j)** bending initiated hinge-terminating fracture on proximal extremity (x8.4); **k)** large amount of blood residue and animal tissue (x200, dark field); **l)** proteinaceous residue including blood (x500, dark field); **m)** proteinaceous residue with blood (x500, dark field).

**Fig 21:**
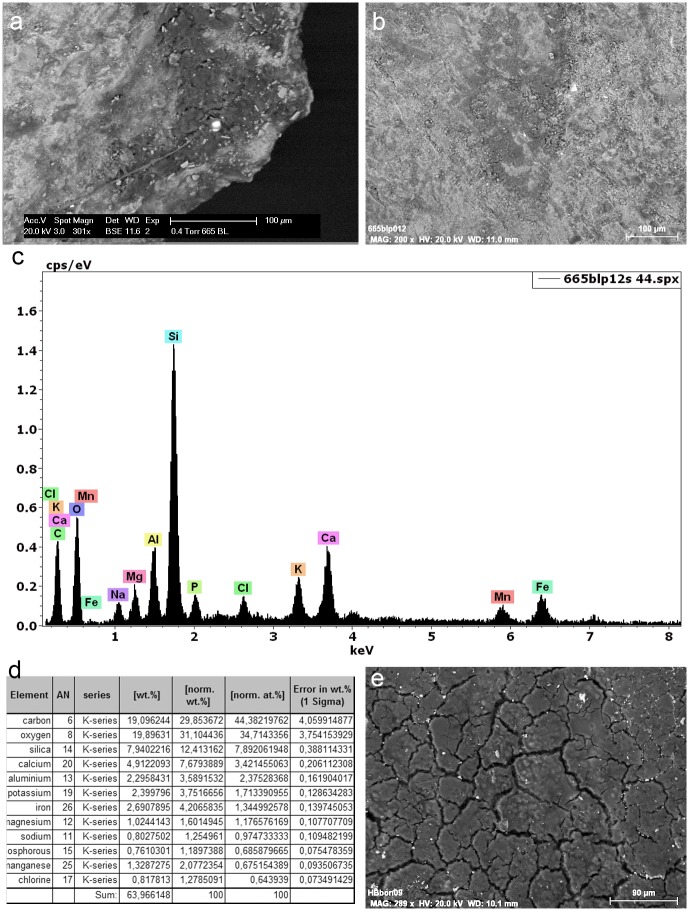
Blood residues on point 665. **a-b)** SEM images of cracked residue cf. blood on the dorsal distal tip (a) and on the dorsal left distal margin (b); **c)** EDS characterisation of the cracked residue showing elemental composition of the distal margin residue with high levels of oxygen and carbon and levels of iron, phosphorus, sodium and chlorine which concur with blood; **d)** table with EDS analytical results; **e)** cracked blood residue from modern comparative reference material.

**Fig 22:**
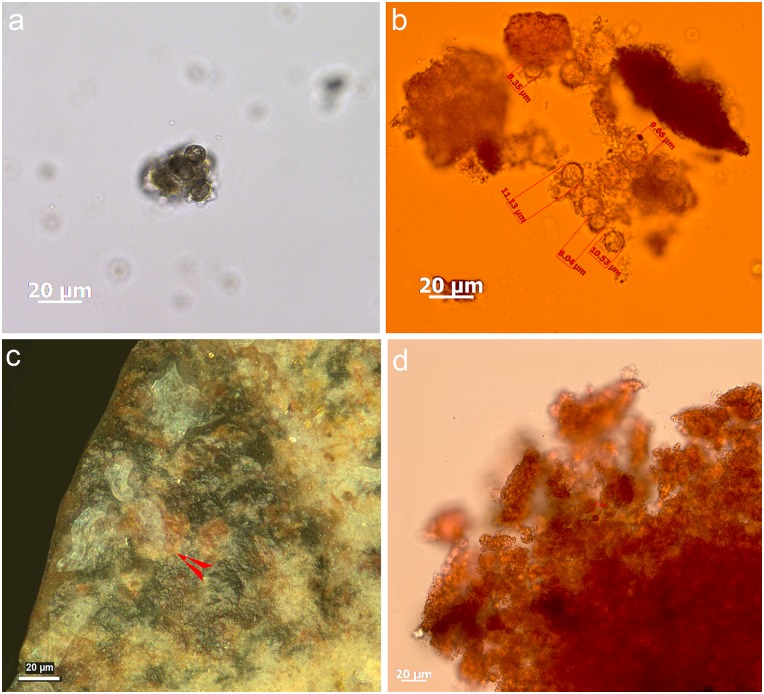
Blood cell residues. **a)** cf. white blood cells (leukocytes) from the dorsal distal surface of 875 (x400, transmitted light); **b)** leukocytes from rock hyrax reference material stained with Eosin (x400, transmitted light); **c)** arrow points to RBC-like inclusions in residue on the ventral left distal to medial edge of 875 (x500, bright field); **d)** hyrax blood sample stained with Eosin (x400, transmitted light).

Convincing evidence of projectile use also comes from an abundance and broad array of well-preserved animal residues prevalent on all the examined unbroken pieces, as well as three distal fragments (Table 10; Fig 19). These consist of smears of proteinaceous residue on distal tips and distal to medial surfaces in association with torn fragments of dermis/hide, torn muscle, fibres, sinew, hair, fat, bone, blood and vivianite. An exceptional example of preservation is shown on the distal to medial surfaces of 665 (Fig 20). Thick residue on the distal tip of this piece was characterised by SEM-EDX analysis as having high levels of carbon, nitrogen and phosphate, confirming that it was proteinaceous residue (Fig 21). This was in association with sinew and large splotches and runnels of blood residue on the left dorsal distal to medial edge and distal to medial surfaces (Fig 20). Proteinaceous residue and blood-like residue associations were also observed on a number of other tools (including 1136, 551, 875, 535 and perhaps also 877 and 845). Red blood cell (RBC)-like inclusions on 1136 and 551 had diameters of ca 5 μm. The residue extracted from the distal tip of 875 which has granulate leukocyte-like inclusions with diameters >9 μm is possibly a white blood cell cast (Fig 22a and 22b). Similar to the calcium carbonate crystals found in the sediment samples, this may derive from rock hyrax (Fig 22a and 22b). As discussed previously, such casts are typically from urine and it is possible that this residue is not use-related but comes from the sediment. However, another blood-like residue on the ventral left distal to medial edge which does appear to be use-related comes from the same point 875 (Fig 22c). This residue had RBC-like inclusions with diameters of 5 μm. Notably, this is within the range of RBCs from several animal species including hyrax as well as zebra and impala [65] (Fig 22d). Coincidentally, a torn hair fragment in the pipette extraction from the distal tip is comparable with horse hair and impala but does not conform with hyrax. This particular point may thus have been used for hunting large mammal species, possibly zebra, which is the only known endemic *Equus* species of South Africa, or one of the Bovidae species which includes impala. However, this evidence should be treated with caution as one hair fragment with poorly visible hair scales is not sufficient for a positive identification. Further chemical analyses may provide a more definitive answer.

**Fig 23:**
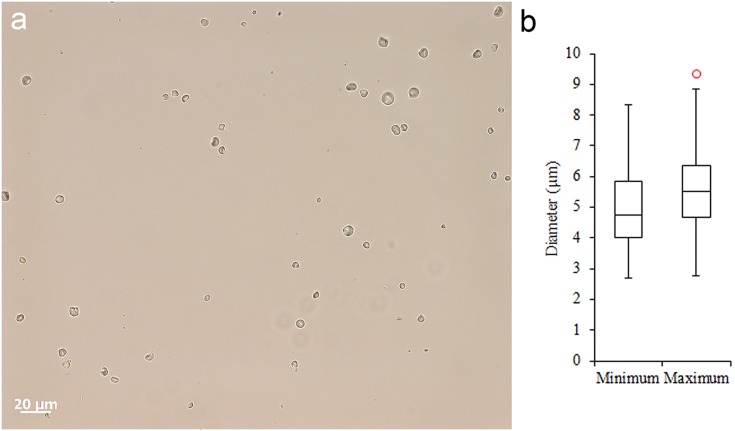
Starch residues. **a)** abundant small starch granules with centric hila extracted from the dorsal distal tip of 1075.1 (x400, transmitted light). These were possibly derived from a plant extract used for poison; **b)** boxplot with minimum and maximum dimensions showing the general roundness of starch granules.

Another interesting question regarding possible use-related residues comes from the abundance of very small to small sub-round and polygonal starch granules recovered from the tip of the small quartz distal fragment 1075.1. The mean minimum and maximum diameters of granules were 4.98 ± 1.01 and 5.57 ± 1.08 respectively (N = 50) (Fig 23a and 23b). There are several possibilities for the provenance of this starch, ranging from contamination from post-excavation handling or the sedimentary context, contact with plant material perhaps from missing the target or from protective covering or sheath of starch-rich plant material, or from a starch-rich extract such as plant-derived poison. Contamination from handling or from the sedimentary context is the least likely source. Not only was starch extremely rare in the associated sediment sample, but these tiny and abundant granules were shown to be very well adhered to the stone surface. Granules could only be removed with ultra-sonication. Such strong adherence also makes it unlikely that the starch is the result of accidental contact with plant material during its use-life (cf. [66]). Likewise, the contact with plant material from missing the target is improbable, as the point is only a small distal fragment and its presence at the site can only be explained by it being brought back inside an animal carcass. Previously missed shots can of course not be excluded. Even though the argument is inherently weak, the poison hypothesis is a distinct possibility and should not be excluded. Identification of the starch and/or other examples of similar starch assemblages on tools may lead to a more definitive answer.

### Evidence of hafting

Evidence of hafting was observed in the form of wear traces and residues in the proximal parts of several pieces. Hafting wear evidence primarily consists of scarring, although some limited polish, rounding or linear features occasionally occur (Figs 15–17 and 20). The wear evidence is suggestive for the occasional use of bindings combined with resin. Hafting residues include resin remains and possible fibres used to fix the stone points in their shaft.

**Fig 24:**
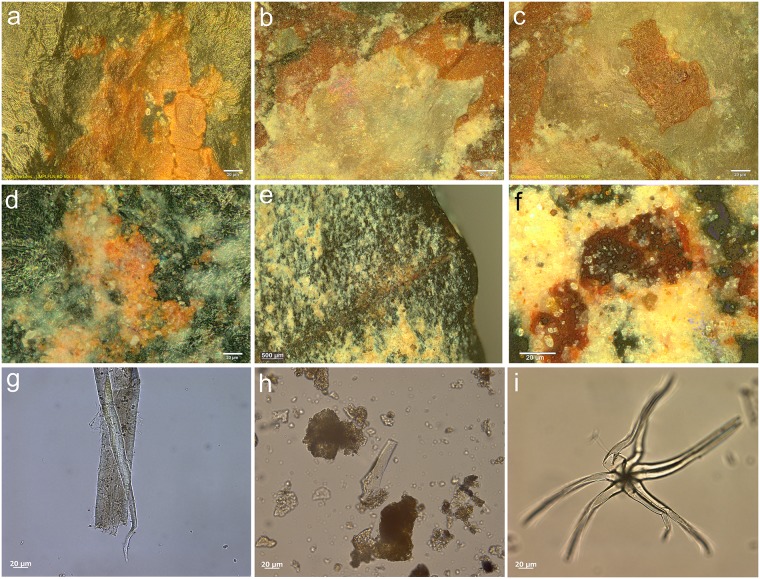
Examples of hafting residues. **a–c)** film of cracked compound adhesive residue on the dorsal distal to medial surface of 1804 with sheets of sinew covering the adhesive residue (b) and fat (c) (x500, dark field); **d)** fatty red-orange pigment on the dorsal proximal surface of 1021 (x500, dark field, polarisation); **e)** red/orange lineal residue on the ventral left medial edge of 551 is possibly associated with binding material (x50, bright field); **f)** cracked red-brown resin on the dorsal medial surface of 632 (x1000, bright field, polarisation); **g)** fibre from the medial surface of 1803 possibly derived from binding (x400, transmitted light); **h)** example of a large vessel element phytolith from the proximal surface of 877 and possibly derived from a wooden shaft (x400, transmitted light); **i)** stellate trichome cf. *Pavonia* sp. from the proximal surface of 1170 (x400, transmitted light).

**Fig 25:**
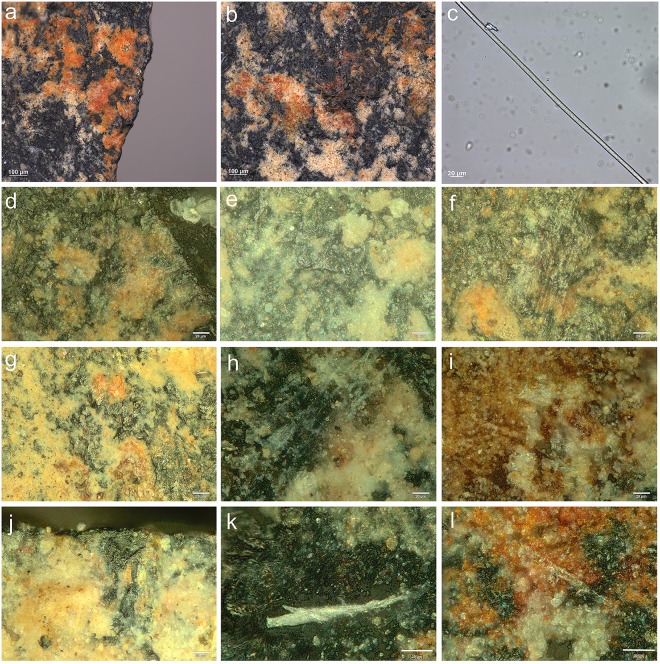
Residues on serrated point 1021. **a)** distribution of red-orange residue, possibly adhesive residue on the dorsal right proximal edge (x180); **b)** fatty residue with red-orange residue (possibly adhesive) on the dorsal left proximal to medial surface (x180); **c)** hair fragment in pipette sample taken from the ventral and dorsal surface of the distal tip fractures (x400, transmitted light); **d)** fatty proteinaceous residue on the dorsal distal tip (x500, bright field); **e)** curled fibre embedded in residue on the dorsal distal surface (x500, bright field); **f)** oblique lineation with fatty and red-orange residues on the dorsal distal surface (x200, bright field); **g)** red-orange residue possibly blood on the dorsal left medial surface (x200, bright field); **h)** articulated connective tissue with fat globules on the dorsal central distal to medial surface (x500, dark field, polarisation); **i)** connective tissue with red-orange residue on the dorsal central medial surface (x500, dark field, polarisation); **j)** lineal striations associated with fatty proteinaceous residue on the dorsal right distal tip (x500, bright field); **k)** twisted animal fibre on the ventral central distal to medial surface (x1000, dark field); **l)** sinew-like fibre in red-orange residue on the ventral right medial surface (x1000, dark field).

**Fig 26:**
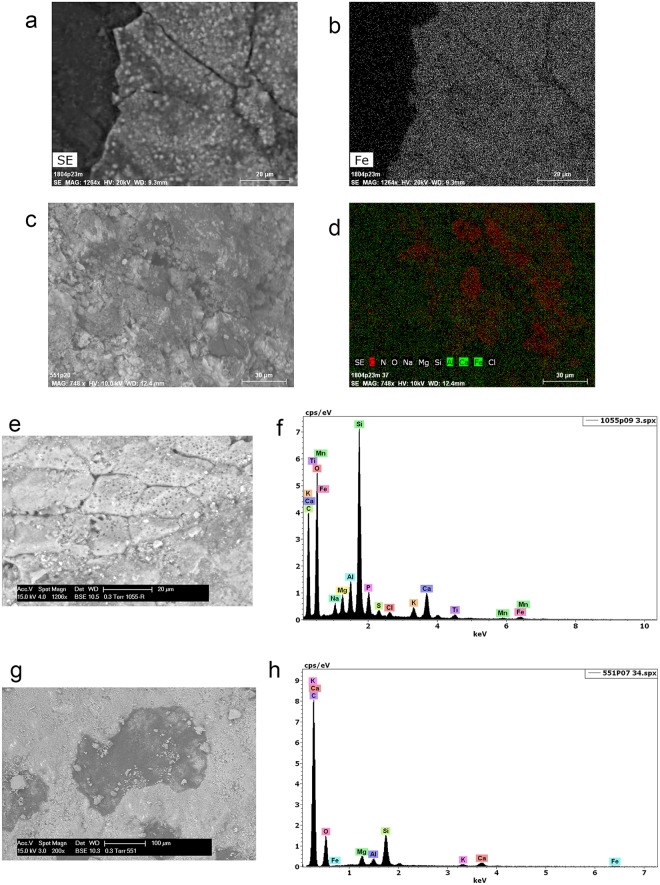
Examples of hafting residues. **a and b)** SEM images of adhesive residue on the dorsal medial surface of 1804 clearly show iron-rich particulates probably derived from ground ochre; **c and d)** SEM images of resinous lump on the dorsal right medial edge of 551 showing distribution and concentration of carbon and other elements including iron; **e and f)** SEM image and EDS spectra of resinous wood on the ventral medial to proximal surface of 535; **g and h)** SEM image and EDS characterization of the black carbon-rich residue found distributed over the surface of 551.

**Fig 27:**
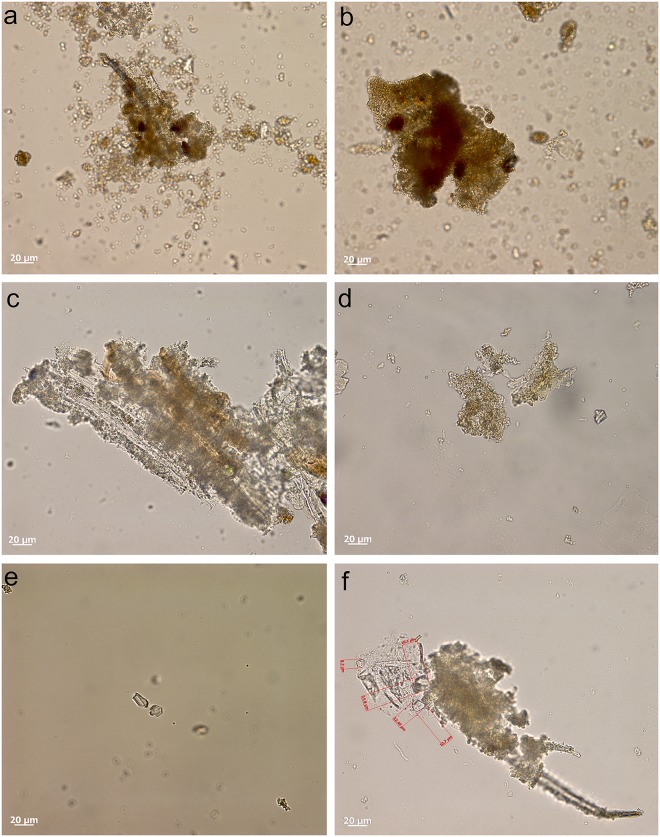
Examples of *Podocarpus* residues and comparative plant material. **a and b)** cf. *Podocarpus* sp. tissue recovered from the medial and proximal surfaces of 1136; **c–f)** an example of a polygonal starch granule from the proximal surface of 1170 (c) in comparison to articulated fibres (d), granular tissue (e) and starch granules (f) from *Podocarpus falcatus* stem reference material (x400, transmitted light).

Traces of residues that concur with a suite of potential hafting residues were present on the medial and/or proximal surfaces of all the tools examined and occasionally on the distal to medial surfaces (Table 10, Figs 24–26). Residues from nine pieces included resin, starch granules, torn plant tissue fragments, fibres and phytoliths that are all present in *Podocarpus falcatus* wood (Fig 27) and are very likely to be derived from an adhesive made from this species or closely related species. The attribution to *Podocarpus* is based on the co-occurrence of micro-fossils: starch that occurs in *Podocarpus* (often a range of morphotypes occurs), tissue fragments with the same cellular arrangement as *Podocarpus*, fibres and phytoliths. With the possible exception of tissue fragments with isometric crystals, which are strongly diagnostic, none of these micro-fossils in themselves are specific to the genus. However, when they occur all together on one tool, or frequently, across an assemblage of tools, they can be relevant and strongly diagnostic, as is the case here. This interpretation based on morphological analysis is supported by previous studies using chemical analysis of residues on MSA stone tools from two other South African sites, DRS (ca. 56 ka; [67]) and Border Cave (ca 43−42.5 ka; [68]). At those sites, plant resins were analysed by GC-MS and identified as *Podocarpus*. Furthermore, another two tools from Sibudu with ages of ca 65 ka and ca 62 ka have also been analysed chemically and retain traces of resin identified as conifer, most likely from *Podocarpus* sp. [56,69].

**Fig 28:**
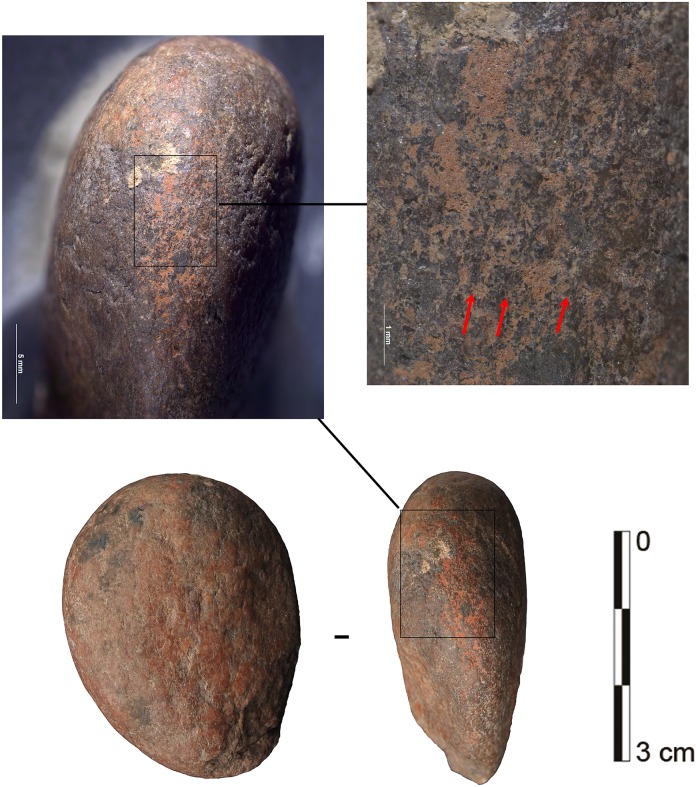
Cobble with ochre staining (layer Chantal).

Some of the resinous residues occur with fat, micro-charcoal, long strands of plant tissue and red ochre/hematite, collagenous tissue sheets and sinew (Table 10; Fig 24). Thick deposits of hematite, indicative of hafting residue, occur on the medial and proximal surfaces of a dolerite piece (1021; Fig 25e). Fatty deposits as well as a tracheid, a twisted plant fibre associated with a large medial fracture scar could also be hafting residues. The most clearly portrayed example of a compound adhesive in this study comes from the medial section of the distal quartz fragment 1804 (Fig 24a–24c). The SEM micrograph shows fine granular particles distributed evenly within the carbon-rich matrix of the resin (Fig 26a and 26b). Coupled with this, the EDX elemental spectra (Fig 26a and 26b) show a very strong correlation between these particles and iron and oxygen, confirming them to be iron oxide, otherwise referred to as hematite, red ochre or red pigment (e.g., [55,56,69,70]). The evenness and small diameter of particle sizes suggests that the iron oxide material was finely ground before being added to the resin. SEM and EDX analyses also confirm iron oxide in association with carbon-rich resinous residues on the left medial to proximal surface of the dolerite piece 535 (Fig 26e and 26f) and the right medial margin of 551 made from hornfels (Fig 26c, 26d, 26g and 26h). Notably, a small cobble with applied hematite from use as a grinder/polisher, recovered from layer Chantal (Fig 28) is supportive of ochre being ground at the site. Perhaps ochre was ground merely as a colorant but its primary function may have been as a loading agent (see [55,71,72]).

It appears that sinew, dermis and plant tissue were part of the fixation agent or that bindings may have been used in combination with the resin. In some cases large sheets of sinew were clearly compressed along and around edges. However, the extensive spread of animal residues upon impact could account for this and the attribution of animal residue to the potential use of bindings is difficult to ascertain (cf. [57]). The resinous compound on 1804 is covered in a large sheet of sinew-like connective tissue and fat globules which also occur close to the edge on the dorsal left medial surface (Fig 24b). Such a distribution is more suggestive of usage rather than binding. Collagenous tissue also occurs on the dorsal right proximal edge of 1170, but again, its origin is likely to be from contact with the bone compressor (cf. Figs 10 and 15). Nevertheless examples of lineal deposits of resinous tissue and sinew-like connective tissue which wrap around medial edges of tools (e.g., 551 and 877, Figs 24e and 17) are more likely to be linked with the use of bindings. Long sinew fibres in association with resinous particles were also recovered, but only in the ultra-sonic extractions, so it is difficult to ascertain their cause. Plant fibres were even more difficult to discern in-situ, but we can note their presence in pipette and USB extractions; many sources (including contamination from the sediment) are possible. However, the presence of other haft elements such as woody fibres in resinous compounds, wood fragments, large robust, vessel element phytoliths probably from wood, hair and large fragments of skin tissue are testimony to other haft elements such as wooden shafts and perhaps even sheathing made from hide.

Of special interest with regard to possible shaft or binding residues is the small serrated quartz piece 1170 (Fig 15). Numerous stellate trichomes were recovered from medial and proximal pipette samples and one fragment was recovered from the proximal USB residue sample (Fig 24i). Also a small fragment of fibre possibly derived from a stellate trichome was found in the USB sample from the medial section of the dolerite piece 1136. These types of trichomes are very common in leaves and stems of Malvaceae and are quite possibly derived from a plant species within this family. *Urena lobata* L. and *Pavonia* spp. are likely candidates. Although the *Pavonia* genus is endemic to the African continent, *Urena* is thought to be derived from Asia, but this is not known for certain [73]. Nevertheless, plants within both genera are very similar with the same shrubby habit and both have a strong baste that is easily stripped from stems and commonly used for cordage [74]. Importantly, the wood is light and strong and the straight stems of one species found in Namibia, *Pavonia schimperiana* Hochst. ex A. Rich., are known to have been used for arrow shafts [75]. Therefore, baste from a species within this plant group was possibly used for binding or stem was used for the shaft.

The size of the points, in particular their width and thickness, is interesting, and has special implications with regard to hafting. Maximum widths vary between 13 mm and 39 mm for all complete points. Only one quartz point is complete (width 13 mm), but based on at least one projectile base fragment in quartz, a width up to 17 mm is possible. For dolerite, widths vary between 14–39 mm for complete points; for hornfels, maximum widths vary between 23–28 mm, for the quartzite point the maximum width is 18 mm. These maximum widths are relevant in view of the maximal width of the shaft on which these points can potentially be mounted given that their morphology and wear pattern dictates that they would have been mounted in a terminal axial position on the shaft. A shaft end width cannot be wider than the point width as it would stop the projectile from intruding into the animal. To have an even more accurate estimate, the width at the base is also relevant. For quartz, the basal width varies between 9–12 mm, for dolerite between 15–24 mm, for hornfels between 18–22mm. This suggests that either two shaft types and potentially different shaft widths may have been used for the quartz points versus the other points, or one shaft width of about 12 mm was used for all points. The point thickness is also interesting in this regard. While the length and width of the points may vary, their thickness is relatively constant and averages around 8 mm (min. 6 –max. 10 mm) for complete points, independent of raw material. Hence, there seems to have been a conscious attempt to reduce point thickness, suggesting that only one shaft diameter was used. The point thickness is indeed important with respect to hafting, for the shaft diameter and the haft type (juxtaposed or split). Both size parameters indicate that shafts of maximum 12 mm in diameter were probably used. This fits remarkably well with the stem size of the Malvaceae shrubs, *Pavonia* and *Urena* spp.

## Discussion

### Using pressure flaking to serrate

Pressure flaking has long been purported to originate as recently as ca 25–20,000 years BP in the Solutrean context [76–78]. However, a recent study on the SB assemblage of Blombos Cave has demonstrated its use during the last stage of shaping, as long ago as ca 75 ka BP in South Africa [5]. The technological analysis supplemented with experimental data showed that pressure shaping occurred together with heat treatment of the silcrete raw material to create straight and regular tip sides. The pressure technique is also claimed to be employed on different raw materials in the SB assemblage of Hollow Rock Shelter [79], while at other SB sites, such as Diepkloof, no evidence of pressure flaking has been recognized [30].

At Sibudu, pressure flaking was previously indicated for the HP layers of Sibudu Cave based on the recovery of bone compressors [59] and of quartz bifacial points [80]. However, it is believed to be absent in the SB assemblage [81,82], where the shaping of the bifacial pieces was done with soft direct percussion.

More recently, Högberg and Lombard [7] have found indications of pressure flaking at Umhlatuzana, a site ca 90 km from Sibudu. The authors published their analysis on a set of serrated points, unifacially or bifacially shaped, older than 70 ka and associated with the SB. Interestingly, they recognize the use of pressure at different stages of the reduction sequences and, consecutively, for different purposes. Their set of evidence suggests pressure was used to shape the preform, to finish the bifacial points and also, to serrate their edges, though there is a need for future experimental and quantitative morphometric analyses [7].

In this context, our study of the serrated pieces of Sibudu provides more evidence for the timing and functional range of the use of pressure in the southern African MSA. Our study is based on multiple lines of evidence (positive, negative and remnant). It shows that the serrated bifacial points of Sibudu Cave have been manufactured by pressure with bone compressors. This is not only indicated by the presence of fragments of bone compressors found in association with the points, but also by a very strong body of evidence from technological as well as microscopic wear and residue analyses that bear witness to the contact with bone on the edges of tools within the concavities of retouch negatives. Hence we can conclude that the pressure technique was used to serrate edges of bifacial and unifacial points and that it is older than 77 ka in South Africa.

At Sibudu, we have not clearly recognized other evidence supportive of a broader use of pressure. However, we acknowledge that the use of pressure might have been employed variously at the last stage of the shaping, for example, to prepare platforms, to shape the points and, of course, to serrate the edge. In other words, we see no contra-indications of a broader use of bone compressors while shaping a bifacial piece. The use of pressure has technical advantages. It markedly diminishes the risk of failure by having more accurate contact points (priority is given to precision and not to force) and thereby it reduces the risk of breakage. Indeed, though pressure in the hand does not avoid the risk of breakage, the use of such a technique diminishes the vibration waves that expand through the blank, which are often regarded as the main cause of breakage during shaping. We acknowledge that the knappers of Sibudu, though their first intention might have been to serrate edges, might have benefited from the other advantages offered by this technique. Further analysis will provide more information in this regard. One advantage associated with pressure lies in its potential to shape new designs and create these with more consistency.

### Serrated points for hunting

Though the tool production clearly aimed at triangular elongated pieces with serrated edges, we observed an internal variability in the population of serrated pieces from Sibudu. The toolmakers did not use specific original blanks to further shape them into the desired tools. The selection of raw material however, had a great influence on the end-product. For all rock types except quartz, we identified two *chaînes opératoires*: bifacially shaped blanks that have been bifacially serrated and bifacially (or unifacially) retouched blanks that were serrated. The pieces made from quartz only correspond to the former described *chaîne opératoire*. Moreover, fewer accidents during the serration procedure seemed to have occurred on quartz, as all pieces are finished and show traces of use apart from one specimen. In addition, the raw materials also have considerable size variation. The quartz points are generally smaller than the other rock types relative to the size of the original chosen blanks, but potentially there is also variation in relation to use and hafting.

Our evidence shows that the serrated points have been used as hafted elements in hunting weapons. An association of impact-related wear features and animal residues were observed on the used portions of the points, while hafting-related wear traces and residues were identified on the non-active tool portions. These observations push back the age of hafted projectiles, identified based on functional evidence, for South Africa. Previously, hafted projectiles were identified based on wear and residues for the HP and post-HP MSA of Sibudu Cave [55,56,69], while all other known evidence is based on fracture patterns only, some of which (i.e., Kathu Pan; [13]) is believed to be unreliable [14]. The basal layers of Sibudu remain undated, but an OSL age of 77.3 ± 2.7 ka BP was obtained for the overlying layer BS [25] implying that the serrated points are older.

Hence, there is growing evidence for hafted projectiles in South Africa of similar ages to other areas, such as Northern Africa [83,84] and the Near East [85]. Functional evidence for earlier hafted projectiles has been documented for Europe (e.g., [86]), even though one has to acknowledge the low frequency of identified projectiles per site in comparison to the tool frequency and assemblage size. The new evidence from Sibudu makes a significant contribution to the scenario that organised hunting with this kind of specialised tool-kits formed a standard and important element of subsistence strategies from at least ca 80 ka BP onwards.

### Hunting with points mounted on shafts

Evidence for hafting of the serrated points is equally strong, especially for the use of a compound adhesive for mounting the points to their shafts. The adhesive seems to be similar to that identified by Wadley and colleagues [87] for the HP layers of Sibudu Cave. This extends the use of such compound adhesives to far beyond the HP. While wear and residue evidence of hafting indicates a much earlier appearance of hafting around 200 ka BP in most areas (e.g., [86,88]), evidence of adhesive use is relatively rare. Earlier (non-compound) adhesive use was observed in Europe. In the case of Campitello, Italy, it appears that pitch may only have formed a wrapping around the tool since no evidence for an attachment to a handle was observed [89]. Pitch also appears to have been used for hafting armatures and other tools at Inden-Altdorf, Germany, but no details on the use-related wear or residue evidence or the overall trace patterning were presented along with the pitch evidence [90]. Strong evidence was however presented for the use of bitumen as hafting adhesive at Umm el’Tlel, Syria, where it dates to about 70 ka BP [91–93].

It has been inferred based on the location and orientation of the functional evidence that the serrated points would have been mounted as tips on the extremity of a shaft. This is further supported by their symmetrical morphology and shape. An end-on position implies that their size provides conclusive information about shaft widths that were used. Based on the measured widths and thicknesses of the serrated points, it is inferred that a shaft type with a maximum diameter of 12 mm was probably used. The predominantly narrow stems of the shrubby Malvaceae species (*Urena* and *Pavonia*) definitely fit with these criteria and their use as projectile shafts has been documented ethnographically [75]. Being relatively short, they would only be suitable for arrow shafts. Sturdier stems, more suitable for large tools and longer shafts, would have had to come from other species. Unfortunately, there is no convincing diagnostic residue evidence for any particular species that might have been used in the case that longer shafts were employed. The robust vessel elements found on the two similar-sized basal quartz fragments, 877 and 1803, occur in several plant groups, and although small globular phytoliths typical of Euphorbiales (e.g, *Macaranga* spp.) occur in medial and proximal residues of another slightly larger quartz piece 632, they also occur in distal residues and in the associated sediment samples and may therefore be derived from the sedimentary context rather than a wooden shaft. Given that one of the smallest and one of the largest and widest tools in the assemblage have residues suggestive of being hafted to the short stems of a Malvaceae shrub, it is conceivable that all the pieces in the assemblage were similarly hafted and used in the same manner.

The proposed shaft widths should of course be dealt with critically since widths are not necessarily continuous over the total shaft length; shafts are often barrelled in shape being narrower at both extremities. In that case, the basal widths of the serrated points are only indicative of the distal width of the shaft used. On the other hand, the point size should be large enough to pierce the hide of an animal sufficiently to also allow for the insertion of the shaft. In addition, if shaft extremities are too reduced, it will affect the sturdiness of the weapon.

### Stone-tipped shafts to project

An intriguing aspect of these points is of course their projection mode. The presence of bow-and-arrow technology has been argued for the HP layers of Sibudu [15], but this interpretation was based on circumstantial evidence only. Indeed, in spite of attempts based on, for instance, TCSA values ([94]; but see [95,96]), no reliable reference framework is currently available to identify projecting modes and it is still unclear whether functional data could succeed in providing one (cf. [48]). For the demonstration, the argument of the authors was mainly based on evidence by association i.e., the existence of strings (for beads) which combined with the faunal evidence was used to argue for the presence of snares. The potential existence of ‘know-how’ for snares was subsequently used to argue for the possible existence of ‘know-how’ to produce bows. The proposed existence of bow-and-arrow technology was derived from this assumed ‘know-how’ in combination with functional evidence on quartz segments that would indicate their mounting as transverse weapon heads. Transverse mounting of the quartz segments is based on the orientation of the damage and striations assumed to be linked with impact [69]. However, the depicted striations (cf. [69]: Figs 5 & 6) are natural surface features within the quartz and do not support any specific orientation in the shaft. The argument for mounting of arrows is based on the above reasoning as well as the fact that their small size would potentially allow it. In contrast, the other segments of the HP layers were manufactured out of other raw materials and were generally larger and variable hafting positions were proposed based on functional data [56]. In this context, the evidence of the serrated points brings an interesting element to discussions about projecting modes. While the HP segments seem to indicate a possible lateral mounting [56,69,97], the serrated points, by contrast, were definitely mounted as tips and not as barbs. Their size is thus an essential element in the discussion regarding projection modes.

Ethnographically, point sizes and projection modes are variable and depend on the hunting context itself: solitary hunters or group hunting, dangerous or docile prey, small or large prey, solitary animals or herds, etc. Nevertheless, some tendencies can be observed. First, thrusting spears need to be distinguished from all other projecting modes. Thrusting spears may be thinned extensively at the tip in order to accommodate for the hafting of small points. However, in a hunting context, small points mounted on thrusting spears will only be able to pierce a hole without creating a lot of bleeding. This implies that small points should be combined with barbs in order to create a sufficiently severe haemorrhage. Within our studied collection from the layers containing serrates from Sibudu, we currently have observed no evidence of barbs. Secondly, the different projected armatures need to be distinguished. Hand-delivered spears can be excluded: spears would need to be significantly reduced at the tip to allow hafting of the serrated points, which is unlikely because at the moment of impact, flexion occurs and if the shaft is too thin it may fracture. In addition, their velocities are relatively low implying that the hafted stone point needs to be large in order to create a sufficiently severe wound and enough bleeding. A minimum of 26 mm for the shaft diameter at 15 cm from the tip was identified in TraceoLab experiments, 15 cm being the minimal insertion of the shaft in a reasonably sized animal to have some haemorrhage. A stone point with a width of 12 mm would thus not allow the insertion of the shaft up to 15 cm. For the serrated points, therefore, assisted projection is likely to have been used. Three options are: (1) spear/arrow shafts that were delivered with a flexible spear-thrower, involving a simple cord twisted around the shaft and used to project the weapon similar to how a sturdy spear-thrower in wood or hard animal material would be used but at lower velocities. None have been documented for Africa, but this is of course also due to the poor chance of cord being preserved. However, based on available data for spears (drawings from ethnography (e.g., Polynesia, [98]) and practices in the Antique World) this technique is only used in combination with heavy spears (2.5–4 kg). For arrows, the technique is currently still used for hunting small game (cf. “flêche Suisse”) as a more straightforward hunting technique than with bows. Velocities are slightly higher than for hand-delivered arrows, so points need to be sharp enough to pierce the hide; (2) thin spear shafts delivered with a hard spear-thrower even though none of those have yet been recovered for Africa in spite of the excellent organic preservation at many sites; or (3) arrows delivered with a bow. In our opinion, scenario 1 (arrows) and 3 are best supported by the different lines of evidence. A bow is definitely one of the most viable options given the small size of the quartz points and the relatively limited diameter of the deduced shaft widths (12 mm max). However, it remains a fact that identifying projecting modes should be based on larger samples and extensive and systematic experimentation [48].

### Projecting one weapon type

The question remains whether the size difference between the quartz points and the points made from other raw materials reflects different weapon types. Ethnographically, it has been documented that different projecting modes may co-exist, for example, a thrusting weapon and a projected weapon (cf. Kalahari San, [99]). Generally, the thrusting weapon is used to kill off, similar to how contemporary hunters finish the kill with a hunting knife. While we acknowledge the likely co-existence of different projecting modes, it is hard to argue that the small set of serrated points would include different projection modes, in particular given that other–not yet functionally examined–bifacial points exist within the assemblage. One projecting mode is thus likely, but it may have been used to shoot different types of stone-tipped shafts [100], each with their own purpose: (1) a lighter weapon with smaller quartz tips intended either for smaller game or for piercing the hide and perhaps deliver a poison; and (2) a heavier weapon with larger points for larger game or for intruding deeper into the animal and cause severe haemorrhage. Game size is probably not a factor as it has been observed ethnographically that stone-tipped projectiles are almost exclusively used on large game (+40kg) ([101]: 40). By contrast, it is evident that the use of poison has a crucial effect on hunting strategies and weapon design. Possible evidence of poison was observed on one of the quartz points. Indeed, abundant plant material was identified on the tip of point 1075.1 that could potentially come from a poison (alternative explanations such as contact with a protective sheath proved unlikely). However, poison use cannot be excluded for the other raw materials. In the case of Sibudu, it therefore seems probable that the different point sizes did not affect the shaft size and projecting mode. After all, the point thickness remained relatively constant between raw materials and thickness is a crucial factor in hafting and use. Moreover, it is a recurrent feature at Sibudu that quartz tools are smaller than tools made from other raw materials, without necessarily affecting function.

### The serrated bifacial pieces from Sibudu within a regional and temporal perspective

The new assemblages unearthed at Sibudu fuel the discussion on the temporal and spatial distribution of the bifacial phenomenon during the southern African MSA [6,30,82,102]. Field results show that the bifacial technology at Sibudu has a longer duration than the chronology conventionally accepted in the literature ([25,103] but see [104]). The new evidence from Sibudu as well as that published in recent papers [30,82,102] urge caution about viewing MSA bifacial technologies in general and the SB in particular as an homogenous technological tradition. In addition, the results from Sibudu demonstrate that the bifacial phenomenon is not a continuous phenomenon as the layers immediately below the SB (layers LBG) are said not to contain any bifacial pieces [2,27].

While the affiliation of the serrates layers within the SB techno-complex may be disputed, it is worth noting that the layers of Sibudu that include the serrated pieces differ in many ways from the overlying SB, though the toolmakers used the same spectrum of raw materials with a clear preference toward dolerite. The main differences between the two relate first to their reduction sequences as the SB is largely dominated by bifacial reduction sequences, with bifacial shaping flakes that compose 91.8% of the lithic assemblage and only a very low proportion of cores [82]. This is also illustrated by the proportion of bifacial pieces that account for 53.1% of the SB while they represent only 21.4% in the ‘serrates layers’. Furthermore unlike the ‘serrates layers’, the SB knappers rarely shaped quartz into bifacial pieces while we have shown that quartz had a peculiar place within the functional system of the knappers responsible for the assemblage in the ‘serrates layers’. Finally, no serrated pieces occur in the SB assemblage of Sibudu. In sum, besides the common presence of a bifacial reduction sequence, the SB and ‘serrates layers’ seem to have little in common and the question of their technological affiliation should be considered with caution.

The set of serrated bifacial pieces discovered at Sibudu increase the range of morphological variability within the MSA bifacial pieces. Presently, similar specimens have only been documented in the sequence of Umhlatuzana, but also, a few specimens appear to occur in the so-called ‘Rhodesian Still Bay’ [105–109]. At Umhlatuzana, the serrated points occur in association with Still Bay points as well as in a level underlying the SB without diagnostic characteristics of the SB. The serrated points have been interpreted as a localised variation of the Still Bay Industry [16]. At Sibudu, the serrated points are systematically found in layers underlying the SB. So far, and due to the insecure stratigraphic context of Umhlatuzana [16,110,111], we contend that the sequence of Sibudu should be taken as a reference with regard to the nature and form of bifacial expressions in KwaZulu-Natal. As a consequence, we suggest that no definite evidence currently exists for serrated bifacial pieces during the conventional time period associated with the SB.

Morphologically, the serrated points of Sibudu Cave are less elongated than those of Umhlatuzana and the serration on the Sibudu points appears to be overall less indented (for comparison see [7,16]). However, Umhlatuzana and Sibudu serrated pieces share similarities with regard to the preferential raw materials that were used as well as to their diversity in shaping, as both assemblages are variously composed of bifacial, partially bifacial and unifacial points. Morphologically, published data [16,102] suggest some similarities between the bifacial pieces of Sibudu and those of Umhlatuzana, notably regarding the morphology of their bases. Finally, dimensions of the serrated pieces are similar between Umhlatuzana and Sibudu supporting the idea that they were both made by fine pressure with a bone tool compressor.

Our results concur with other authors [6,30,82,102] that are inclined to see the MSA bifacial technologies not as one single and unvarying technological expression but as a broader technological phenomenon encompassing more variability in space and potential differences in time. In that respect, the new set of bifacial pieces from Sibudu substantially change the perception we had of MSA bifacial technologies in the southern African MSA, expectedly and assumedly of a relatively short-term existence. Sibudu, along with evidence from elsewhere, including that from the Pietersburg (see [112,113] for Border Cave; see [34] for Bushman Rock Shelter), show that the bifacial pieces find earlier expressions than those associated with the SB. The serrated pieces from Sibudu present a clear and strong techno-functional signature with regard to other technological MSA manifestations presently known. Based on currently available data, the assemblage of the ‘serrates layers’ seems to have little to compare with the SB as it is presently known at Sibudu, not only on the basis of the serrated bifacial pieces but also on the basis of a specific laminar reduction sequence which is being analysed currently. The lithic technology of the ‘serrates layers’ of Sibudu, together with the stratigraphic evidence from the site, offers the unique possibility to evaluate its relation with other assemblages with bifacial tools and we consider the possibility that they should not be integrated within the SB techno-complex (largely defined on the presence of foliate or lanceolate points and semi-circular or wide-angled pointed butts [103]) but rather be viewed as a new technological phase. Further studies and analyses will confirm or refute this proposition.

### The serration, as a solution for what?

Serrated bifacial points occur much later in other areas. Different types of serrated points are known from the Paleoindian period of California and the Southwest of the USA [114,115]. Another example comes from the Kimberley Region of Northwest Australia, where so-called pressure flaked Kimberley points occur around 1.4 ka BP [36,63,116,117]. One other example is given by the serrated ‘*pointes aveyronnaises’* from the Chalcolithic context of the *Grands Causses* Region [118]. One interesting aspect from the literature is that most of the serrated points, which often have specific names, seem to be restricted in time/duration and space/diffusion [114,118,119]. Further studies are needed to better evaluate the geographical and temporal range of distribution of the serrated bifacial points within the southern African MSA.

Based on the present literature, the role of the serration on the points has different interpretations. Many authors have suggested that the serration was not necessarily orientated towards function and that such pieces represent a specific design related to specific contexts such as burials or ceremonies [114], or used as trade goods [36], designed to reflect individual ownership or tribal affiliation [120] and as active symbols of social membership [119]. The main demonstration of these possibilities comes from recent experimental work published by Loendorf et al. [119], who observe no clear relationship between the role of the serration and functional attributes such as accuracy, wound size or durability, suggesting to them that the serration may not be related to the point function. However, a few authors acknowledge that the serration might represent ‘barbs’ [114] or functional characteristics [121,122] that might have improved the efficiency/penetration of the hunting weapon. It has also been observed that serrated points create terrible wounds [101,123] and that they are often associated with animals regarded as being dangerous or warfare.

There is a substantial variation within the set of published serrated points, in terms of morphologies and dimensions (of the blanks and of the notches) as well as potentially, of purposes. The serrated pieces from KZN, both from Sibudu Cave and Umhlatuzana, offer such examples of inter- and intra-variability. Unlike published and later specimens (though there is some internal variability in the types published: see [114]), the serrated points from KZN have shallower serrations. The significance of this difference should be first discussed from a functional perspective.

The analysis of the Sibudu points does not allow a definite conclusion on the functional significance of the serration, but some hypotheses can be proposed. The serration increases the contact area of the point with the animal target upon insertion, which may increase the haemorrhage and bleeding of the animal. While bifacial retouch reinforces the edges and makes them more resistant to damage during projectile impact, it also reduces the cutting capacity of the edges. The serration is perhaps a way to compensate for this loss by again increasing the damaging effect of the edges upon impact, which may lead to an increase in penetration [122]. It may also be an advantage in the case of bone contact. This hypothesis only works if the point tip is sufficiently narrow as to affect the shape of the cross-section. As argued, pressure allows working points differently with an effect on the transversal cross-sections that can be obtained. Hence, it allows for an emphasis on the point section, not on the edge. Finally, we have also raised the possibility of poison being used. One could argue that the serration was intended to retain the poison. Alternatively, a combination of multiple reasons may apply.

## Conclusion

Based on a detailed study of the technological characteristics of the points, the shaping flakes, the wear and residue evidence and the recovery of bone compressors, we show that the bifacial serrated points from the lowermost deposits (Adam to Darya) of Sibudu Cave were notched by pressure flaking. This pushes back the date of the oldest evidence of pressure flaking that was until now associated with the SB context of Blombos Cave dated to ca. 75 ka and with the points from Umhlatuzana said to be older than 70 ka. In addition, the data from Sibudu Cave confirm that the pressure technique may be applied for different production stages, either shaping as demonstrated at Blombos Cave or serrating as it has been proposed at Umhlatuzana.

This technology and the set of serrated tools find no equivalent in the present record with the exception of the site of Umhlatuzana. Unlike Sibudu, the pieces from Umhlatuzana are argued to be associated with the SB techno-complex [103]. By contrast, the extensive and careful excavation of the well-stratified deposits of Sibudu currently seems to indicate that serration did not occur at the site after 77 ka. We argue that the sequence of Sibudu should be taken as a reference for the KZN MSA and that the association of the serrated pieces with SB layers dated to 73–71 at Umhlatuzana may be clarified by renewed excavation at the site.

The excavation of the layers containing serrated points of Sibudu Cave helps to complete our picture of the succession of technological phases that occurred in KwaZulu-Natal and South Africa. Similar to the Pietersburg Industry, our data show that bifacial technology is not restricted to the SB: it actually appears earlier in time and has punctuated manifestations. The implications of these new assemblages of Sibudu Cave within the context of the chrono-cultural framework of the southern African MSA suggest that the bifacial manifestations were diverse and autonomous in South Africa. The data from Sibudu indicate that the bifacial phenomenon represents several temporal and/or regional phases.

The serrated pieces from Sibudu Cave introduce new insights in the significance of MSA bifacial technologies. In the SB of DRS for example, the bifacial pieces have been interpreted as cores and mobile tools that were (re-)sharpened from time to time up until their discard (Porraz et al., 2013), within the framework of populations with a fair degree of mobility. The SB assemblage of Blombos Cave indicates that the on-site manufactured bifacial pieces were used as spear tips for hunting [124], while in the SB of Sibudu Cave, the bifacial pieces represent, in most cases, long-lived cutting tools through re-sharpening as well as reworking and sometimes tips of hunting weapons [82,125]. The bifacial points of the layers with serrates of Sibudu reveal a different picture, with serrated bifacial pieces being finished tools used for hunting. The evidence illustrates the multiple facets of bifacial technology and the diversity underlying their broad technological definition. Sibudu Cave provides the unique opportunity to continue exploring the diversity of bifacial strategies, to evaluate its implications for other bifacial assemblage types and to assess chrono-stratigraphic relationships.

In addition, the wear and residue evidence shows that these bifacial points were hafted with a compound adhesive as tips on projectile shafts and that they were used to hunt animals. This evidence pushes back the date of projectile points identified based on wear and residue analysis for South Africa to a date older than 77 ka BP. While the functional evidence allowed the identification of the serrated points as projectiles, their exact projecting mode is more difficult to interpret. Nevertheless, there are distinct arguments in favour of bow-and-arrow technology or, alternatively, perhaps flexible spear-throwers (cords).

The serration itself is a functional innovation that is presently unique in the MSA. Such functional implements have been documented in much more recent contexts, for instance, during the Holocene in Western Europe, the Paleoindian period of North America and the last millennium in Australia. While denticulates and notches are common modifications on tools, the serration itself seems to be restricted to few contexts and can be regarded as a strong techno-cultural marker.
